# Implementing One Health governance approaches to mitigate antimicrobial resistance across institutional, social, economic and political contexts: a scoping review

**DOI:** 10.1136/bmjopen-2025-115471

**Published:** 2026-07-08

**Authors:** Chloe Clifford Astbury, Taylor Hecker, Jaskeerat Singh, Suzanne Garkay Naro, Lauren Wallace, Geneviève Boily-Larouche, Mathieu J P Poirier, Yahaya Ali Ahmed, Anand Balachandran, Zlatina Dobreva, Walter Fuller, Laetitia Gahimbare, Yidnekachew Degefaw Mazengiya, Bassem Zayed, Arne Ruckert

**Affiliations:** 1 York University, Global Strategy Lab, Toronto, Ontario, Canada; 2 School of Global Health, York University, Toronto, Ontario, Canada; 3 World Health Organization Regional Office for Africa, Brazzaville, Congo; 4 World Health Organization, Geneva, Switzerland; 5 World Health Organization Regional Office for South-East Asia, New Delhi, India

**Keywords:** Systematic Review, Health policy, Public health

## Abstract

**Abstract:**

**Objectives:**

Antimicrobial resistance (AMR) poses a serious threat across human, animal and environmental health. The One Health approach emphasises multisectoral collaboration and is critical in addressing AMR. While One Health governance has gained recognition from international organisations, there remains limited understanding of how it can be effectively implemented across institutional, social, economic and political contexts. This scoping review aims to explore the design and implementation of One Health governance across contexts.

**Design:**

Scoping review

**Data sources:**

We searched PubMed, Scopus, Web of Science and grey literature sources in December 2024, updating our search in March 2026.

**Eligibility criteria:**

Eligible sources included empirical and conceptual work on One Health governance.

**Data extraction and synthesis:**

We searched for and screened documents and extracted data following Preferred Reporting Items for Systematic Reviews and Meta-Analyses Extension for Scoping Reviews (PRISMA-ScR) guidelines. We then applied qualitative analysis to examine governance mechanisms; implementation contexts; conceptualisations of effectiveness; evidence of effectiveness and key barriers and facilitators.

**Results:**

We included 171 documents from over 50 countries. We explored six dimensions of One Health governance: participation, leadership, coordination, decision-making, resourcing and accountability. Existing governance structures and wider aspects of country context shaped One Health governance. While there was broad agreement on the goals of One Health governance—namely, to support disease prevention, detection and response—empirical evidence on effective approaches was relatively limited. Facilitators included strong political will and crisis-driven momentum, while barriers included siloed systems, sectoral dominance, limited accountability, inadequate funding and lack of institutionalisation.

**Conclusions:**

This review highlights the range of approaches to One Health governance that exist and outlines how context may shape the design and implementation of One Health governance. Evaluative research should further explore which approaches to One Health governance are most effective in specific contexts. These insights are particularly relevant for AMR, where sustained cross-sectoral governance beyond outbreak-driven responses is essential to counter the ‘silent pandemic’.

**STRENGTHS AND LIMITATIONS OF THIS STUDY**

- The qualitative analysis was performed using a hybrid deductive-inductive approach that allowed results to be built from the existing evidence and include novel findings.
- The scope of the search was wide and included information from over 50 countries across all seven World Bank regions and a range of income levels.
- Literature searched and included was limited to documents in English or French; this may have introduced a degree of selection bias since some documents, especially policy documents, may have been published in national or local languages only.
- This review highlights mechanisms, contextual factors and current barriers and facilitators to the implementation of One Health governance.

## Introduction

 Antimicrobial resistance (AMR) is a global threat that undermines efforts to treat infectious diseases effectively across human, animal, plant and environmental health sectors.^1 2^ Addressing this complex issue requires a One Health approach, which promotes multisectoral and multidisciplinary coordination and collaboration across these domains. Developing a strong One Health governance framework is critical to enabling cross-sectoral collaboration.^3^ The Quadripartite, comprised of the WHO, the Food and Agriculture Organization, the World Organisation for Animal Health and the United Nations Environment Programme (UNEP), has committed to strengthening cooperation through One Health to tackle challenges like AMR.^4^ However, despite this commitment, challenges in implementing One Health governance at the national level across varied institutional, social, economic and political contexts remain, including how to best integrate One Health governance within the existing governance landscapes.

In this review, we define One Health governance as the structures, processes and mechanisms that enable intersectoral coordination, collaboration and shared knowledge and decision-making across the human, animal, plant and environmental health sectors to sustainably balance and optimise the health of people, animals and ecosystems. This also includes commitment to key normative principles required for successful One Health implementation, including transparency, sustainability, inclusivity and equity.^5 6^

The existing literature highlights several key factors important for successful implementation of One Health governance models, such as multisectoral coordination mechanisms, political commitment and adequate resource endowment.^7^,^9^ However, evidence of how different countries have approached designing and implementing One Health governance has not been comprehensively mapped. As a result, existing recommendations do not provide guidance on how to navigate context-specific barriers such as, for example, designing multisectoral coordination mechanisms in federal systems; agenda-setting for One Health where there is a lack of political commitment; or implementing One Health activities funded by external donors.

Beyond this, the development of evidence-informed recommendations for effective One Health governance has been hampered by a lack of theory and evidence about what works. In this review, we define ‘performance’ as the extent to which One Health governance achieves its stated aims. Demonstrating performance for One Health governance first requires a clear conceptualisation of what these aims are. Existing definitions have included process-oriented aims, such as strengthening systems or improving collaboration; outcome-oriented aims, such as securing ‘optimal’ outcomes, improving outcomes relative to what might be achieved by working in sectoral siloes or improving cost-effectiveness; and normative aims, such as improving equity, inclusion or sustainability.^5 10 11^ Clarifying the definition for performance of One Health governance and understanding whether and how this has been measured would support the development of a more robust evidence base to inform recommendations.

### Aims and scope

Taken together, an understanding of different approaches and how they perform in different contexts can support countries in developing and implementing effective One Health governance. The objective of this scoping review is to synthesise what is currently known about implementing One Health governance in diverse institutional, social, economic and political contexts. This will include mapping the range of One Health governance mechanisms that have been implemented in specific contexts; evidence of performance within those contexts, including how performance has been defined and assessed; and barriers and facilitators to implementing One Health governance across different contexts. Given the cross-cutting nature of One Health governance structures and the possibilities for shared learning, our review includes evidence from other domains that require One Health governance (eg, zoonoses, vector-borne diseases).

## Methods

We conducted a scoping review on the context-specific implementation of One Health governance mechanisms, as described in our previously published protocol.^12^ This scoping review was conducted in accordance with guidelines published by Arksey and O’Malley and refined by Levac *et al*.^13^,^15^

This scoping review is reported in accordance with the guidelines outlined in the Preferred Reporting Items for Systematic Reviews and Meta-Analyses Extension for Scoping Reviews (PRISMA-ScR).^16^

### Stage 1: identifying the research question

This review aimed to map and analyse the implementation of One Health governance mechanisms across diverse institutional, social, economic and political contexts. Guided by our aim, the research questions were:

1. Which One Health governance mechanisms have been implemented within or across specific contexts, and which stakeholders have been involved?
2. How has the performance of One Health governance been conceptualised and measured in the literature, and which mechanisms are associated in the literature with success and failure within specific contexts?
3. What are the barriers and facilitators to implementing One Health governance within and across these contexts?

### Stage 2: identifying relevant studies

To ensure the interdisciplinary breadth and depth of the sources covered, we searched three electronic databases (PubMed, Scopus and Web of Science) on 13 December 2024 and performed an updated search on 23 March 2026. The search strategy was organised by the main concepts in our research questions: One Health; infectious diseases and governance (online supplemental file 1). We developed the search strategy iteratively, informed by previous reviews on related topics^8 17^ and indicator papers known to the authors as meeting inclusion criteria.

We also searched for grey literature, using domain-specific desktop searches of relevant organisational websites (online supplemental file 2).

### Stage 3: study selection

Records found through the searches were compiled and screened using Covidence.^18^ We screened titles and abstracts followed by full texts for inclusion based on the criteria in table 1. For both title and abstract and full-text screening, two reviewers screened independently, and conflicts were resolved by consensus in discussion with a third reviewer.

**Table 1:** Inclusion criteria

Criteria	Inclusion criteria
Publication date	2000–March 2026 (present; a period encompassing the first uses of the term ‘One Health’ by international organisations in the early 2000s through to more widespread use and application in subsequent years^10^)
Geographical range	Any country or region
Thematic focus	Documents that refer to any of the following issues: One Health governance implementation in the context of specific institutional, social, political or economic realities. Barriers (eg, infrastructural) and facilitators (eg, cultural adaptability) to implementing One Health governance models in different contexts. The performance of One Health governance models in the context(s) in which they were implemented.
Language	Only articles in English and French will be included
Type of literature	Documents including empirical evidence and/or theoretical or conceptual developments, of any of the following type: Peer-reviewed articles. Reports from governmental, intergovernmental (eg, WHO, FAO) and/or non-governmental organisations.

FAO, Food and Agriculture Organization.

### Stage 4: charting the data

We extracted key characteristics from the included documents:

- Lead author or publishing institution; year of publication; country focus.
- Region using World Bank classification.^19^
- Country income using World Bank classification.^19^
- Data collection period.
- Data sources.
- Study aim.
- Overall topic characterised as One Health (where focus of governance was not pathogen-specific), AMR, neglected tropical diseases, avian influenza or other.

We qualitatively analysed the included documents using a hybrid deductive-inductive approach.^20^ We started with an a priori coding framework (online supplemental file 3) based on our research questions and previous theoretical frameworks focused on One Health governance, AMR governance and complexity in policy processes.^8 9 21 22^ The existing frameworks had not explicitly considered context. As a result, we aimed to develop a framework to illustrate how context shapes One Health governance, understanding context as the variation in institutional arrangements, actor configurations, power relations and socioeconomic and political conditions within which governance processes are embedded and enacted. Our analysis builds on contextual aspects that have been considered in previous scholarship, namely formal aspects of governance such as political structures, informal aspects like power dynamics and informal relationships, and aspects of wider country context like economic drivers.^10^ We then refined and expanded the coding framework inductively through analysis of the included documents. All qualitative coding was conducted by one of a team of coders (CCA, TH, JS, SGN, LW) using MAXQDA.^23^ Before starting to code, all coders completed test coding of 3–5 documents, which were reviewed and discussed with the lead author (CCA) to ensure that the concepts in the coding framework were well understood. Throughout the coding process, coded material was reviewed and discussed iteratively within the research team to ensure conceptual clarity and consensus across coders.

### Stage 5: collating, summarising and reporting the results

We used descriptive summaries of the characteristics of included documents and narrative and tabular syntheses of qualitative findings to answer our research questions. In addition, we compared findings across income groups and governance structures to identify patterns of contextual influence.

### Patient and public involvement

This scoping review is part of a larger project exploring context-specific implementation of One Health governance and involves a steering group made up of WHO staff members from headquarters and African and South-East Asian regional offices. Input from this group has informed the development of this study, and some group members have contributed as coauthors.

## Results

### Sample characteristics

After removing duplicates, our search identified a total of 2145 peer-reviewed academic records and 1019 grey literature documents (online supplemental file 4). After screening, 171 documents (148 peer-reviewed and 23 grey literature) met our inclusion criteria (online supplemental file 5).

**Figure 1:**
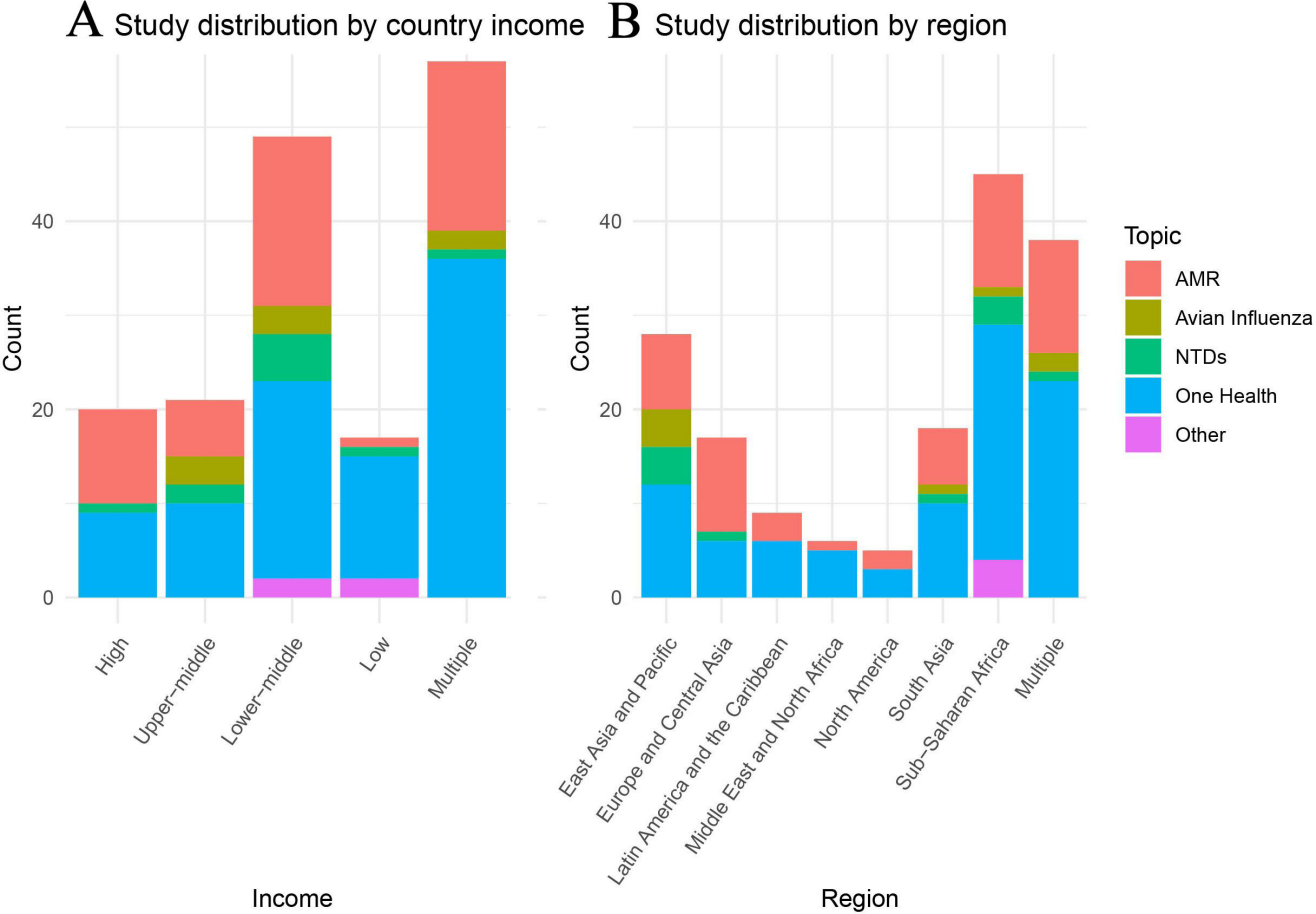
Study distribution by income (A) and region (B), broken down by topic (n=169). Theoretical studies were excluded where they did not refer to any specific country context (n=2). AMR, antimicrobial resistance; NTDs, neglected tropical diseases.

Many of the included publications included data from multiple countries (n=63) and the remaining focused on 48 separate countries. Publications focused on multiple income settings (n=57), high income (n=20), upper middle income (n=21), lower middle income (n=49) and low income (n=18) (figure 1). Geographically, most publications were focused on Sub-Saharan Africa (n=45), East Asia and Pacific (n=28) and South Asia (n=18), with many encompassing multiple geographical regions (n=38) (figure 1). Literature focused on One Health (ie, not pathogen-specific, n=92), AMR (n=56), neglected tropical diseases (n=10), avian influenza (n=8) or other (n=5, included pathogens such as malaria or anthrax). Studies were published between 2008 and 2026.

**Figure 2:**
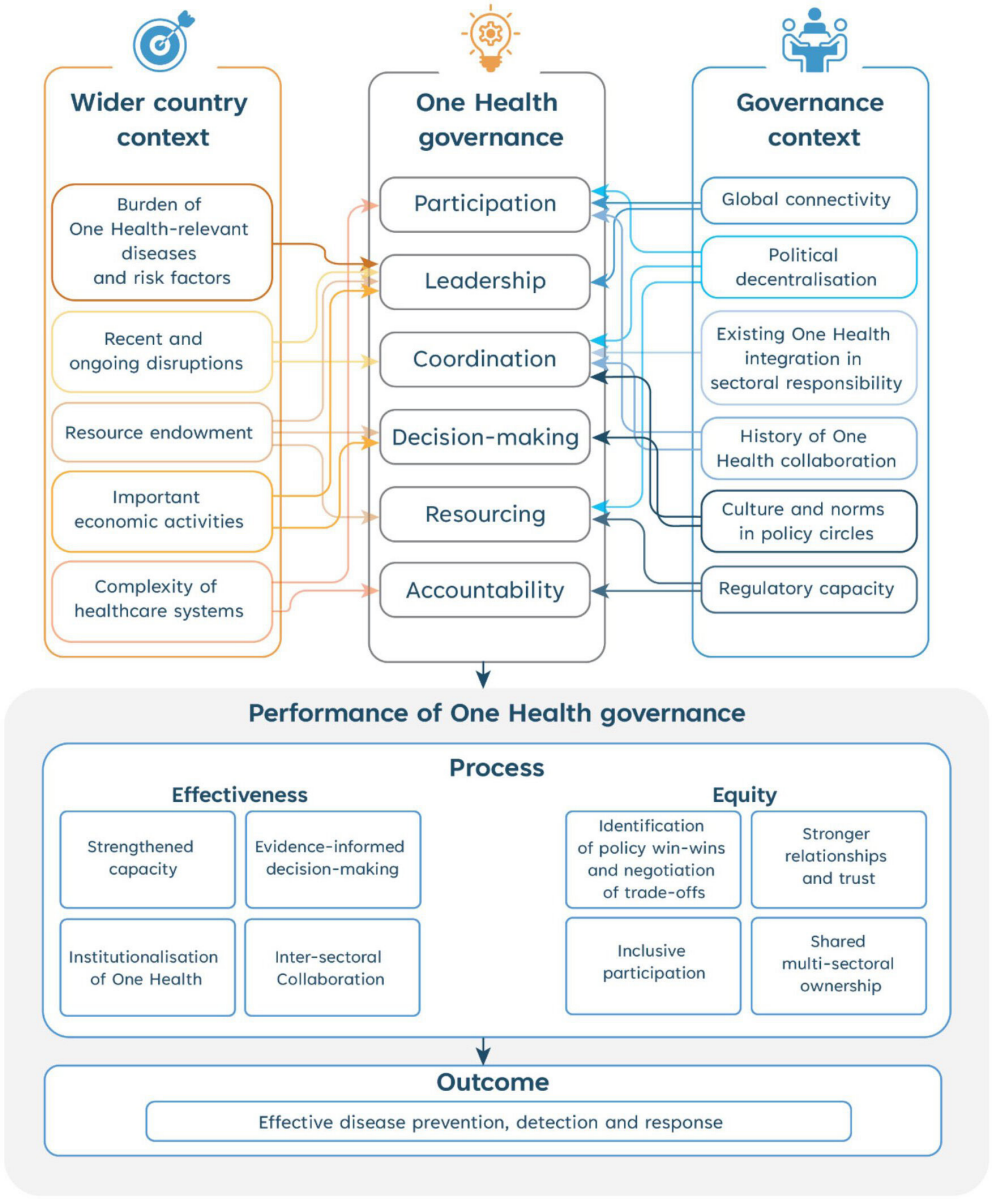
Overview of key contextual factors, One Health governance mechanisms and conceptualisations of performance. Arrows indicate connections supported by theoretical and empirical evidence synthesised in this study but may exclude important connections not documented in the literature.

Through our analysis, we identified a range of contextual factors that shape One Health governance, One Health governance mechanisms and conceptions of effective One Health governance (figure 2). These are described in greater detail below.

### Implementation of One Health governance mechanisms across contexts and actors

We identified six dimensions of One Health governance with significant contextual variation: participation, leadership, co-ordination, decision-making, resourcing and accountability.

### Participation

First, participation focused on who was involved in One Health governance, their formal and informal roles and their level of engagement. The actors identified in studies and their typical roles are summarised in table 2. Overall, not all actors were engaged in One Health governance to the same extent. National governments generally played a central role, and subnational governments could be key actors, particularly in decentralised contexts. Non-state actors were engaged to different degrees, with many studies finding that the general population in particular had not been given adequate opportunities to contribute.^24 25^ Mechanisms to give the general public a voice in One Health governance often included participation of civil society groups, including patient rights groups, community councils and faith-based organisations.^26 27^ Specific venues could provide avenues for civil society organisations to inform policy agendas, such as Thailand’s National Health Assemblies.^28^

**Table 2:** Actors in One Health governance

Actor category	Example actors	Typical role(s) in One Health governance
National government and parliament	Prime ministerial or presidential office; Cabinet; National government departments/ministries (including human health, animal health, agriculture, environment, fisheries, wildlife, parks and conservation, and education); Parliamentarians	Develop and implement policies (eg, AMR National Action Plans (NAPs), One Health NAPs) Develop and implement governance processes and mechanisms (eg, multisectoral coordination mechanisms) Propose and agree new legislation, regulation and memoranda of understanding to facilitate cross-sectoral work Make resourcing decisions for national and subnational One Health activities Lead and participate in multisectoral coordination mechanisms Collect and share data across sectors and governance levels Scrutinise and hold other actors (eg, subnational government, professionals and practitioners, private sector) accountable
Subnational government	Subnational (eg, state, district, city, village) government departments (including human health, animal health, agriculture, environment, wildlife, parks and conservation)	Develop and implement subnational policy Implement policy established by national government Make resourcing decisions for subnational One Health activities Participate in subnational multisectoral coordination mechanisms Collect and share data across sectors and with national-level actors
Professionals and practitioners	Human health professionals, animal health professionals and their associations	Participate in consultations for policy decision-making Implement policies established by government Participate in multisectoral coordination mechanisms Receive training to strengthen One Health capacity
Donors and development partners	Bilateral partners, global coalitions, philanthropic foundations	Provide technical and financial support, with impacts on agenda-setting Participate in multisectoral coordination mechanisms
Intergovernmental organisations	Global (WHO, WOAH, FAO, UNEP); regional (African Union, Association of Southeast Asian Nations (ASEAN), European Union)	Set global and regional norms Provide technical and financial support Participate in multisectoral coordination mechanisms Receive and share data from member states
Private sector	Agricultural sector (smallholders, large-scale farms); pharmaceutical sector; private healthcare sector; representative industry bodies	Participate in consultations for policy decision-making Participate in multisectoral coordination mechanisms Self-regulate Implement policies established by government
Research and educational institutions	Universities (domestic and international); national research institutes; regional university networks; colleges; schools	Develop evidence Provide evidence-informed recommendations Provide technical and financial support Strengthen One Health capacity Strengthen topic-specific capacity (eg, AMR, vector management) Train professionals
Non-governmental organisations	Global (Médecins Sans Frontières, Red Cross), regional (ReACT Africa, Ecumenical Pharmaceutical Network), national (health-focused, faith-based)	Provide technical and financial support Strengthen One Health capacity Strengthen topic-specific capacity (eg, AMR, vector management) Support networking between actors Participate in consultations for policy decision-making Participate in multisectoral coordination mechanisms
General population	Communities, patient groups, consumer groups, youth	Participate in consultations for policy decision-making Receive training to raise OH awareness and change behaviour
Media	Newspapers, television, radio	Participate in multisectoral coordination mechanisms Raise awareness of OH topics

AMR, antimicrobial resistance; FAO, Food and Agriculture Organization; OH, One Health; UNEP, United Nations Environment Programme; WOAH, World Organisation for Animal Health.

The extent of formal involvement of non-state actors in One Health governance also varied depending on the country context. For example, studies focusing on high-income countries (HICs) more frequently referred to the role of the private sector, particularly bodies representing the agricultural^29^ and pharmaceutical^30^ sectors. In low and middle-income countries (LMICs), donors and development partners and intergovernmental organisations were mentioned more frequently.^31 32^ Overall, the concept of One Health is quite well known, and this serves as a facilitator to increased participation; however, in some cases, as in Tanzania, where a robust national coordination system is in place, limited participation and support for local One Health officials can hinder cross-sectoral implementation efforts.^33^

While the literature identified many actors who had formal roles in One Health governance in various contexts (eg, participating in consultations), this did not necessarily imply meaningful engagement, with actors from civil society organisations,^34^ sectors within national government^35^ and subnational government,^36^ at times, seeing the ways in which they had been engaged as ‘tokenistic’. In other cases, although actors had more formal roles, for example, positions on multisectoral coordination mechanisms, they disengaged from participating in these mechanisms, particularly when they were not the leading agency.^36 37^

### Leadership

Second, leadership could be both formal and informal. Formal leadership typically operated by assigning leadership authority to institutions, while informal leadership often came from individuals acting as policy champions.

A key aspect of formal leadership operated through multisectoral coordination mechanisms, which were most commonly led by human health ministries,^38^,^40^ followed by ministries such as agriculture whose remits included animal health.^41 42^ While hosting leadership of multisectoral coordination mechanisms within one of these two sectors was common, this could lead to disengagement from the non-leading sectors and hinder data sharing and coordination.^43^ Some countries opted for other models, such as the Democratic Republic of Congo, where the One Health platform was led by the Ministry for Education as a means to reduce ownership by a single sector, although some stakeholders noted that this ministry struggled to compel other government actors to action.^37^ In several countries, leadership of multisectoral coordination mechanisms was either shared^44 45^ or rotating.^46^ However, challenges did arise. For example, in Uganda, the chair of the One Health platform rotated every 6 months, which was seen by authors as hindering meaningful contribution by the chairs.^25^ Another approach to preventing sectoral dominance in One Health governance was to host leadership of multisectoral coordination mechanisms within an overarching political body, such as a presidential or prime ministerial office.^47 48^

Beyond formal leadership roles, the informal leadership provided by policy champions was frequently discussed. These could include individuals or groups both inside and outside of government. Common policy champions included healthcare professionals,^49^ government bureaucrats^26^ and elected officials.^41^ Policy champions could enable One Health governance and shape decision-making, including by fostering bottom-up approaches, for example, through coalitions led by healthcare professionals,^49^ and by agenda-setting from within government and parliament.^35^ An additional form of informal leadership was provided by donors and development partners, who often played a key role in agenda-setting through their provision of financial and technical support, particularly in LMICs.^34 50^

### Coordination

Third, coordination focused on processes and mechanisms enabling actors to work together. A key form of coordination included the establishment of multisectoral coordination mechanisms. These could either be focused on specific topics (eg, AMR,^51^ avian influenza,^48^ anthrax,^44^ trypanosomiasis,^25^ food safety^45^) or be established to coordinate around One Health broadly, sometimes including subgroups or technical working groups.^37^ These mechanisms could be permanent or ad hoc, the latter often established in response to a disease outbreak.^3^ They were typically established at the national level, which could cause issues for subnational actors when there was a need to coordinate on local issues.^38^ Responses to this included informal collaboration between local actors, as was noted in Burkina Faso,^36^ Fiji^52^ and the USA,^53^ though national actors were not necessarily aware of these connections, or the establishment of more formal subnational One Health platforms, as in the Democratic Republic of Congo.^37^

Beyond multisectoral coordination mechanisms, coordination structures often revolved around surveillance system integration. A review of integrated surveillance systems found that all established country-wide systems operated in HICs,^40^ although studies focused on LMICs identified instances in which data sharing and joint analysis and interpretation had occurred across sectors.^44 52 54^ Another common approach to coordination was through joint planning of work laid out in multisectoral strategies and action plans.^3 45^ A coordination approach that was less frequently undertaken, but often described as important and necessary, was regulatory harmonisation.^3 44 55 56^ Finally, coordination also happened through informal relationships between individuals working in different sectors and using these relationships to build trust.^57 58^

### Decision-making

Fourth, decision-making encompassed approaches across the policy process. Multisectoral coordination mechanisms played an important role and were often established as advisory groups to inform decision-making,^59^ but could also supervise and coordinate activities.^60^ Some countries also had mechanisms dedicated to incorporating evidence into decision-making, such as technical working groups.^59 61^ These groups were sometimes established under the aegis of multisectoral coordination mechanisms. However, uptake of technical working groups’ recommendations by government was not always guaranteed.^59^ Evidence-informed decision-making could also be supported by targeted research and data at certain points in the policy process,^44 62^ such as establishing structured stakeholder dialogue to jointly define and map problems.^63^

Consultative processes also allowed different perspectives to be considered when making policy decisions, including the perspectives of the private sector, healthcare professionals and civil society organisations.^29 59^ Given the wide range of actors involved in One Health governance, power imbalances were often at play, and the perspectives of more powerful actors, both state^42^ and non-state,^64^ could be given greater weight in decision-making. It was not uncommon for the human health sector to view themselves as the leaders or dominate conversations, as was highlighted in a study from Senegal.^55^ One approach to mitigating the impact of power imbalances was to use structured, participatory processes to support decision-making, which were seen as an equitable means to negotiate between different priorities. Key examples of this included cross-sectoral processes to prioritise zoonotic diseases, for which the United States Centers for Disease Control and Prevention’s standardised workshop process was described in several countries^31 65^; processes to select actions for inclusion in AMR NAPs^66^; and International Health Regulations-Pathways for Veterinary Services National Bridging Workshops.^67^

### Resourcing

Fifth, resourcing considerations included where resources came from; the quantity of resources available; and how they were allocated. In HICs, resources almost invariably came from domestic government sources, with the exception of some level of regional support, for example, funding from the European Union for its member states.^68^ In LMICs, models were more mixed, with many countries receiving and relying on resources from donors and development partners.^69 70^ In many cases, this threatened sustainability of One Health efforts,^25 39 65 71^ as in South Africa and Eswatini, when surveillance programmes weakened with the end of donor funding.^56^ The quantity of available resources was almost invariably described as inadequate, particularly in LMICs. In several cases, it was stated that there were resources available to sustain certain One Health governance mechanisms, such as meetings of a multisectoral coordination mechanism, but no resources to implement the activities they were meant to govern.^34 72^ This lack of resources could also result in a lack of capacity to hire or adequately pay personnel, with One Health governance mechanisms being supported exclusively by volunteers^25^ or lacking a focal point to maintain momentum.^3^ This could lead to high staff turnover, resulting in a loss of investment and capacity^34^ and a lack of institutional memory.^36^ Establishing dedicated national One Health focal points or permanent secretariats was identified as a promising mitigation strategy. An additional consideration was around how resources, particularly funding, were allocated, with many emphasising the importance of dedicated financing for One Health activities, while others argued that integrating financing with other planned actions could increase sustainability.^47 62^

### Accountability

The final dimension, accountability, encompassed processes and mechanisms to hold stakeholders accountable for delivering on One Health commitments. An important accountability mechanism was public reporting of monitoring results, including surveillance data^45^ and, less commonly, progress on policy implementation.^62^ This could include reporting to supranational bodies or systems, such as the WHO’s Global Antimicrobial Resistance and Use Surveillance System (GLASS).^73^ While public reporting of spending was not described in the literature, it was advocated for in several contexts.^24 74^ Accountability could also be supported by establishing domestic and international legal requirements.^75^ Context-appropriate regulations can support appropriate antimicrobial use and access to antimicrobials, decreasing the risk of AMR; however, the lack of technical and financial resources can weaken the enforcement of regulations.^76^ Therefore, accountability mechanisms cannot effectively function in a silo. Given the multiple actors involved in One Health efforts, designating specific accountable bodies with clear roles and responsibilities for each planned activity was also a key means of supporting accountability,^37 48^ encouraging actors to consider contributions to cross-sectoral efforts as a core part of their mandate. Finally, monitoring and evaluation mechanisms could also support accountability by tracking policy implementation and outcomes.^33 56^ Overall, no accountability mechanism was seen as a ‘silver bullet’ to ensure policy progress, and the existence of accountability mechanisms that were planned but not implemented,^52^ or were mainly ‘box-checking exercises’,^34 54^ was discussed in several contexts.

### Contextual factors

We identified contextual factors that could shape One Health governance, including both the existing governance context as well as the broader country context (figure 2). These characteristics, and the ways in which they shaped the key dimensions of One Health governance, are summarised in table 3. These findings collectively highlight how contextual variables, particularly political decentralisation, funding architecture and crisis experience, shape the six dimensions of governance.

**Table 3:** Contextual characteristics and impacts on One Health governance

Contextual characteristic	Impact on One Health governance
Governance context	
Political decentralisation (ie, countries with high subnational authority)	Participation: One Health governance is challenging in politically decentralised countries, given the wider range of actors with autonomy and authority that may need to participate in governance structures and processes Coordination: lack of coordination within and between subnational units, including geographically proximate units.^53^ Formal^37^ and informal^36 53^ mechanisms were present in some cases Resourcing: different needs, priorities and resources between different subnational units, and frequent inadequate resources at the subnational level.^37 54^
Existing One Health integration in sectoral responsibility	Coordination: countries with existing integration of One Health-related topics within a single ministry or agency were better positioned to fulfil certain One Health governance functions, like information-sharing and collaborative action. Examples included integration of veterinary and food governance in Norway^45^ or establishment of a Veterinary Public Health Division within the Ministry of (Human) Health in Uganda^25^ 00/00/0000 00:00:00
Culture and norms in policy circles	Coordination: contexts with more informal, relationship-based policy cultures could implement reasonable levels of collaboration without the need for formal structures,^36 38 51 53^ although there were concerns that this was not sustainable given reliance on individual staff members’ connections.^38 51^ Decision-making: hierarchical policy cultures could make it more challenging to implement One Health governance and require more transparent and formalised decision-making processes, as power dynamics in these contexts were exacerbated, for example through tendencies to defer to the authority of human medicine or national-level government.^38^
History of One Health collaboration	Participation: prior history of One Health collaboration, or, in contrast, failure to collaborate effectively in response to past outbreaks, often demonstrated the value of the One Health approach and made actors more willing to engage in it.^3 38 47^ Coordination: in some cases, successful One Health collaboration did not lead to the institutionalisation of One Health, which could be seen as a missed opportunity,^38^ and cross-sectoral collaborations that had been mobilised for some issues, often highly infectious diseases, had not been applied to issues such as AMR.^39^
Regulatory capacity	Resourcing: countries’ capacity to enact and enforce regulations impacted the effectiveness of One Health measures. In LMICs, lack of regulatory capacity was often attributed to lack of funding for enforcement agencies, underlining the importance of adequate resourcing.^34 107^ Decision-making: in HICs, enactment of regulations could be hindered by the influence of powerful private interests, underlining the importance of considerations around transparent and equitable participation and decision-making.^64^ Coordination: in an effort to increase engagement between sectors, some countries, like the Philippines, have begun to bring AMR into other key national strategies (eg, National Action Plan for Health Security, National Environmental Health Action Plan 2030 and 5-Year One Health Agenda) ^108^
Global connectivity	Participation: the extent to which countries were connected to regional and global norms, institutions and markets shaped approaches to One Health governance. Many studies reported the influence of regional and global norms and actors, and the establishment of One Health governance structures and actions that were in line with regional and global guidance, particularly from the Quadripartite organisations (eg, alignment of AMR NAPs with the WHO’s Global Action Plan on AMR), from regional governance organisations (eg, alignment with EU Action Plan on AMR or the Association of Southeast Asian Nations’ Rabies Elimination Strategy), and/or to align with International Health Regulations commitments.^45 90^ Leadership: export to international markets, particularly of livestock products, could drive political will on One Health governance and approaches^48 54^ in order to align with regulations in target export markets. This included where there was otherwise a lack of commitment to agendas such as AMR mitigation
Wider country context	
Burden of One Health-relevant diseases and risk factors	Leadership: the burden of One Health-relevant diseases (eg, rabies, Ebola, avian influenza, AMR) and risk factors (eg, high biodiversity, expanding human-animal-environment interface, injudicious AMU) played an important role in agenda-setting for One Health governance, fostering greater political will to implement One Health approaches. In particular, past spikes or crises (typically outbreaks) could act as important enabling events to prioritise One Health as a political agenda and demonstrate the success of multi-sectoral collaboration.^29 38 48 109^
Resource endowment	Leadership: lack of resources also fostered greater reliance on donors and development partners, impacting leadership structures (eg, with donors and development partners being represented on One Health platforms).^80 98^ Decision-making: reliance on donors and development partners could shape decision-making processes, sometimes privileging global priorities over national ones.^80 98^ Resourcing: countries’ resource endowment impacted various aspects of their infrastructure and capacity, including surveillance and laboratories, communication and healthcare, with implications for willingness and capacity to sustainably implement One Health approaches and make evidence-informed policy decisions^34 39 47 50 110^ 0/0/0000 0:00:00 AM
Recent and ongoing disruptions	Leadership: in many countries, recent and ongoing disruptions, including conflict, global instability, political instability, inflation spikes and economic collapse, could detract from a One Health agenda and cause a loss of momentum and interest in One Health topics.^36 37 44 51 111^ Coordination: in some cases, a history of dealing with ongoing disruptions could make governance mechanisms and actors more adaptive and resilient.^51^
Important economic activities	Leadership: the importance of One Health-relevant economic activities, particularly animal agriculture but also tourism, primary resource extraction and healthcare-related activities such as pharmaceutical production or private healthcare delivery, could impact attitudes to One Health approaches and the perceived importance of tackling One Health-relevant diseases.^39 44 51 107^ Resourcing: the economic importance of these activities could also give relevant private actors outsized influence on policy processes, underlining the importance of transparency and conflict of interest safeguards around participation and decision-making for One Health governance.^100 112^
Complexity of healthcare systems	Participation: human healthcare systems with multiple providers and authorities (national/subnational, private/public) posed challenges for One Health governance given the number of actors whose participation was required.^113^ Accountability: multiple governance mechanisms enabled accountability for these different actors (eg, regulation, peer pressure, trust)

AMR, antimicrobial resistance; HICs, high-income countries; LMICs, low and middle-income countries; NAPs, National Action Plans.

### Conceptualisation of success and failure of One Health governance

We identified process and outcome aspects of how strong performance for One Health governance has been conceptualised, finding overall agreement in the literature (figure 2). First, One Health governance should lead to effective processes. This included improving capacity for One Health activities, particularly surveillance,^77 78^ technical,^79^ research^77^ and institutional^80^ capacities. One Health governance was also seen as having the potential to support evidence-informed decision-making,^81 82^ including through the establishment of technical committees.^83^ Finally, One Health governance was seen as having the potential to enable the institutionalisation of One Health, creating new norms and processes.^83^ In turn, this enabled sustained inter-sectoral collaboration.^83^

Second, One Health governance should support inclusive processes, including by fostering attitudinal change among stakeholders. This could lead to enhanced support for One Health approaches,^84^ a sense of shared ownership^85^ and more holistic understandings of problems.^82^ One Health governance was also seen as something that could facilitate navigating sectoral priorities, including negotiating ‘win-wins’^86^ and trade-offs.^35^ Overall, inclusivity and effectiveness were connected: inclusivity was seen as supporting effectiveness,^87^ as it supported legitimacy, stakeholder engagement, feasibility and appropriateness.

Taken together, these aspects of process were seen as being able to support the expected outcomes for One Health governance: more effective infection prevention, detection and response. One Health governance supported these outcomes by enabling a more preventive approach to public health problems^44^ and cost-effective, harmonised action across sectors.^83^

While there was agreement on how an effective One Health governance system should perform, we found limited evaluative evidence. The conceptualisation of strong performance described above was predominantly articulated as something hoped for from implementing One Health governance. Among the few empirical studies on effectiveness, the evidence took several forms. First, some studies referred to the results of standardised evaluations such as the Joint External Evaluation (JEE), citing results as evidence of a country’s capacity in One Health governance.^88^ However, the idea that JEE results could proxy One Health governance was problematised by one study, which compared JEE results with qualitative insights on One Health implementation and found limited associations between the two.^32^ Another form of evidence presented was the qualitative observations of stakeholders involved in One Health governance, noting success in developing positive cross-sectoral relationships^61^ or insights^62^; or failure to foster shared multi-sectoral ownership^89^ or make adequate progress on One Health policy projects, such as the implementation of AMR NAPs.^68^

A final form of evidence presented was case-based, which provided some context-specific insights. Many cases focused on policy activity during outbreak periods, particularly on timely detection and response. While this form of evidence is promising in terms of informing the development of context-specific recommendations, the current literature has limitations: a small number of case studies included empirical evidence and formal analysis,^50^ but many were provided anecdotally by study authors to illustrate how outbreak response had improved as a result of improved One Health governance.^90^ There were examples of successful establishment of ad hoc coordinating bodies in response to outbreaks across different contexts, including in LMICs such as Vietnam^39^ and HICs such as Canada,^57^ where the additional challenge of a federal system was overcome relatively effectively. A number of cases underscored the role of national and subnational authorities in outbreak response, with studies focused on large and highly decentralised countries such as Brazil^91^ and India^92^ noting how the need to escalate data to the national level for decision-making and cross-sectoral information-sharing could impede the speed of outbreak response. In these cases, subnational multisectoral coordination mechanisms, such as those described as being established in the Democratic Republic of Congo,^37^ could facilitate more timely action. While most of the case-based evidence focused on implementation, others explored different aspects of the policy process. For example, a description of bottom-up efforts by healthcare professionals to establish AMR as a policy priority in Mexico in the absence of strong political will not only highlighted some successes in terms of garnering public and political attention for AMR but also noted eventual loss of policy momentum and failure to institutionalise the AMR agenda into higher level policy structures.^49^ This illustrates the potential of bottom-up approaches where there is a lack of political will to invest in One Health-relevant topics as well as the challenges inherent in these approaches.

### Barriers and facilitators to implementing One Health governance

Several barriers and facilitators were described in the literature. First, studies highlighted resource constraints as a key barrier,^89 93^ particularly in LMICs where they could increase dependence on donors and development partners.^94^ Resource constraints were particularly felt in rural regions where limited access to diagnostic tools may hinder early detection and response efforts.^95^

Second, studies noted the lack of political will as a significant barrier.^96^ However, this term could serve as a catch-all for a more nuanced set of dynamics. Political will could fluctuate over time depending on national priorities, the engagement of external actors, or even the evolution of the evidence base. For example, in countries where economic development was the key priority, the demonstration of the *economic* benefit of One Health approaches was cited as necessary to foster political will and support.^32^

Third, information-sharing is vital to One Health governance, with authors stressing the importance of coherent communication, faster coordination, integrated data collection and cross-sectoral engagement.^38 40 83^ A recurring obstacle to cross-sectoral communication was the lack of a common ‘language’ between sectors, which is critical for cooperation.^97^ Siloed work environments were also cited as impeding communication and may occur as a result of differences in training,^38^ differing goals^39^ or poor understanding of the importance of multisectoral collaboration or how best to support it.^98^

Fourth, a sense of ownership for One Health efforts was noted as an important facilitator.^44 82 99^ Shared ownership could be undermined where One Health initiatives were dominated by representatives from certain sectors.^38^ In addition, if the development of One Health governance was viewed as an outcome of international pressure instead of national need, this could impact the sense of domestic ownership, a dynamic more frequently seen in LMICs with high involvement of donors and development partners.^36 37 41 100^

Finally, the experience of past One Health-relevant outbreaks could also be a facilitator.^50 101^ In such cases, successful multisectoral collaboration during outbreaks aided in improving uptake of One Health governance by demonstrating its value.^36^ Unfortunately, several studies showcased missed opportunities for the institutionalisation of One Health governance after a disease outbreak.^37 38^

## Discussion

### Principal findings

This review identified One Health governance mechanisms that have been implemented in specific contexts and the contextual factors that shape them. This included both existing governance structures and wider aspects of country context. Throughout, One Health governance involved multiple state and non-state actors, operating at local, national and international levels. There was agreement across the literature on what high-performing One Health governance should do, namely support the effective prevention, detection and control of infection. Achieving this outcome required governance processes that were both inclusive and effective. Despite broad agreement on these goals, empirical evidence of the effectiveness of different approaches across specific contexts remained limited.

### Comparison with existing evidence

This review builds on previous scholarship conceptualising One Health governance, focusing on similar domains around coordination and resourcing,^8^ but also considering participation, leadership, decision-making and accountability. This broader scope may explain the inclusion of a wider array of literature than previous reviews. The breadth of studies that were included in this review, as well as the analytical approach, allowed us to map context-specific aspects of One Health governance. While previous scholarship has highlighted that One Health governance solutions must reflect national, institutional and socioeconomic realities to be effective,^6^ and that institutional arrangements should be adapted to specific contexts and display the right ‘ecological fit’,^102^ we provide a comprehensive conceptual ontology of context-specific dimensions relevant to One Health governance effectiveness.

### Implications for policy and practice

Since our review identified contextual factors that could shape One Health governance, context-specific governance challenges call for contextually appropriate strategies. In LMICs, resources were constrained, and donors and development partners frequently made a substantial contribution to One Health governance. One Health was often a high political priority in these settings, due both to the participation of these agencies and the frequently high burden of One Health-relevant diseases and risk factors. In many cases, there were formal mechanisms for coordination in place. Resource constraints could also lead to lower capacity to implement activities. These factors led to a combination of challenges, including maintaining sustainability of One Health efforts; the risk of One Health becoming an excessively top-down agenda; and the lack of contextualisation and awareness of existing governance structures in designing One Health governance. Strategies to mitigate these challenges could include planning to transition to domestic funding for One Health activities. In light of the decline in overseas development assistance, key actors such as the Africa Centres for Disease Control and Prevention^103^ have called for innovation in approaches to financing public health initiatives.

While pursuing these strategies may entail substantial institutional restructuring, they may enable more sustainable and context-appropriate efforts for One Health going forward. Other strategies could include ensuring strong engagement and capacity-strengthening with implementing and local agencies; and more comprehensive assessment of existing governance structures. Multilateral efforts must also play a role: the political declaration of the high-level meeting on AMR adopted by the United Nations General Assembly (UNGA) in 2024 includes a commitment to ensuring that, by 2030, all countries have adequate, sustainable human and financial resources to support the implementation of their AMR NAPs, and to mobilise additional financial resources through existing funding structures.^104^ This includes the Multi-Partner Trust Fund on AMR, which focuses on supporting One Health action on AMR in LMICs, though country contributions have not yet met the call for funding.^105^

Meanwhile, in upper middle-income countries, resources remained constrained, but the external funding available to support One Health governance was often more limited. In some cases, One Health could be a relatively low political priority, due in part to the lesser role of donors and development partners in agenda-setting as well as the salience of other important priorities such as economic development. This could present challenges to maintaining momentum on One Health given resource constraints and lower political will, sometimes leading to a lack of engagement from high-level political leaders, and less frequent establishment of formal coordination mechanisms. In these settings, there were examples of attempts to mitigate this through bottom-up approaches to agenda-setting, which could be supported by broad-based engagement and the training and cultivation of One Health policy champions. In addition, funding a dedicated staff member, such as the AMR focal points established in some countries, could support ongoing momentum and the coordination of a wider set of actors. This is in line with scholarship on network governance, where having a lead actor nominated to drive and coordinate efforts can support more effective collaborative endeavours.^106^

In contrast, HICs saw resourcing coming almost entirely from domestic sourcing. Making efficient use of resources through collaborative approaches was key challenges in HICs, as a lack of shared budgeting remained an issue. In the HIC context, the private sector could be very organised and have an entrenched role in policy spheres. Mitigating the risk of policy capture from the private sector was therefore an important consideration. However, this had to be balanced with ensuring adequate consultation and engagement with private actors given their importance as implementing partners. Strengthening public transparency and managing conflicts of interest were noted as crucial for preserving trust in multisectoral governance. Context-appropriate strategies could, therefore, include planning for shared cross-sectoral budgeting and identification of cobenefits and trade-offs as well for strategic and transparent engagement with private sector actors.

While more evaluative evidence is needed, some of the case-based evidence identified in this review suggests that context-appropriate governance models can lead to better performance. For example, highly centralised approaches focused on national-level collaboration can be less effective in politically decentralised countries, hindering timely information sharing and response. In other contexts where there was a lack of high-level political will to invest in One Health agendas, ‘bottom-up’ approaches led by non-state actors could be an effective means of fostering greater interest, policy ownership and momentum.

Overall, these findings support the importance of context in shaping the design, implementation and effectiveness of One Health governance. National governments, and those providing financial and technical support for government decision-making, should recognise context-specific barriers to effective governance and implement the strategies described above to improve performance.

### Unanswered questions

This review highlighted the need for more robust evaluative evidence, including context-specific evidence. Our overview of the dimensions of One Health governance and the way countries with different contexts have approached the design of One Health governance could support the development of this type of evaluation. Additional causal, mechanistic evidence based on comparative analysis and in-depth case studies would support evidence-informed decision-making. This could include qualitative comparative analysis and case study research based on key informant interviews and document analysis. This future research should focus on filling existing knowledge gaps, such as the lack of understanding of the configurational conditions of effective context-specific One Health governance and AMR policymaker knowledge of successful context-specific governance strategies.

### Strengths and weaknesses of the study

This review took a systematic approach in line with published guidelines.^13 14 16^ It incorporates both peer-reviewed and grey literature in recognition of policy-relevant and practice-relevant topics under study. The hybrid deductive-inductive approach to the qualitative analysis^20^ allowed this review to both build on existing theory and scholarship and identify novel ideas.

This review also has some weaknesses. First, due to the nuanced information that we extracted during coding and the authorship team’s language capacities, we restricted our scope to documents in English or French. This may have introduced a degree of selection bias since One Health governance policy documents and implementation frameworks are often produced only in national or official languages and not translated into English or French. This could have led to an under-representation of institutional mechanisms or governance innovations that are not captured in the Anglophone or Francophone peer-reviewed and grey literature. In addition, language and scope limitations of the grey literature screening may have limited insights into national-level idiosyncrasies that would have been captured with a broader search strategy. Second, our grey literature search focused on the websites of global and regional organisations to maintain feasibility. This may have excluded important national-level documents that could contribute to a more complete picture of One Health governance. We recommend that future research projects expand this scope to include national-level document analysis and extend analysis beyond English and French language documents, using AI-based translation tools that are becoming more reliable and hence widely adopted in academic research. Third, studies originating from LMICs were over-represented in the literature, and we recommend expanding the evidence base through future empirical studies focused on high-income settings. Finally, evidence relevant to One Health governance may not always use the term explicitly. To mitigate this, we searched for terms like intersectoral and cross-sectoral, but relevant documents may still have been excluded due to these limitations.

### Conclusion

This review synthesised a broad evidence base to examine six key dimensions of One Health governance: participation, leadership, coordination, decision-making, resourcing and accountability. We identified both common approaches and context-specific variations. We found an overall agreement on the goals of effective One Health governance, namely inclusive and effective action for infection detection, prevention and control. However, significant challenges remain, particularly in securing sustainable financing, overcoming sectoral siloes and institutionalising governance mechanisms beyond outbreak periods, which is particularly important for AMR as a ‘silent pandemic’. The findings underline that governance effectiveness is shaped not only by the mechanisms employed but by how they interact with political, institutional and economic contexts. As such, future efforts must prioritise context-appropriate approaches to strengthen One Health governance globally. As AMR continues to pose a transboundary and cross-sectoral challenge, embedding effective governance within AMR strategies can significantly enhance their sustainability and impact.

## Supplementary material

10.1136/bmjopen-2025-115471 online supplemental file 1

10.1136/bmjopen-2025-115471 online supplemental file 2

10.1136/bmjopen-2025-115471 online supplemental file 3

10.1136/bmjopen-2025-115471 online supplemental file 4

10.1136/bmjopen-2025-115471 online supplemental file 5

## Data Availability

All data relevant to the study are included in the article or uploaded as supplementary information.
