# Supplementary material for: Implementing One Health governance approaches to mitigate antimicrobial resistance across institutional, social, economic and political contexts: a scoping review
Source: BMJ Open. 2026 Jul 8;16(7):e115471. doi: 10.1136/bmjopen-2025-115471 (PMC13347904; doi:10.1136/bmjopen-2025-115471)
Supplement: online supplemental file 1 [file bmjopen-16-7-s001.docx]

**Supplementary file 1**

*Academic database search strategy*

**Scopus (Title abstract keywords)**

*One Health*

"One Health" OR multisectoral OR multi-sectoral OR cross-sectoral OR intersectoral OR inter-sectoral

AND

*Infectious diseases*

amr OR "antimicrobial resistance" OR abr OR "antibiotic resistance" OR "antimicrobial stewardship" OR "antibiotic stewardship" OR "drug resistance" OR "anti-parasitic resistance" OR "antiparasitic resistance" OR "anti-fungal resistance" OR "antifungal resistance" OR "anti-malarial resistance" OR "antimalarial resistance" OR zoono* OR "infectious disease" OR "infectious diseases" OR pandemic W/5 prevention OR pandemic W/5 preparedness OR pandemic W/5 response OR vector w/5 disease OR vector w/5 diseases OR "neglected tropical disease" OR "neglected tropical diseases" OR "food safety"

AND

*Governance*

governance OR regulatory OR “coordination mechanism” OR “coordination mechanisms” OR "policy process" OR "policy processes" OR "policy framework" OR "policy frameworks" OR "policy mechanism" OR "policy mechanisms"

**PubMed (Title-abstract)**

*One Health*

"One Health"[Mesh] OR "One Health" OR multisectoral OR multi-sectoral OR cross-sectoral OR intersectoral OR inter-sectoral

AND

*Infectious diseases*

"Antimicrobial Stewardship"[Mesh] OR "Drug Resistance, Microbial"[Mesh] OR amr OR "antimicrobial resistance" OR abr OR "antibiotic resistance" OR "antimicrobial stewardship" OR "antibiotic stewardship" OR "drug resistance" OR "anti-parasitic resistance" OR "antiparasitic resistance" OR "anti-fungal resistance" OR "antifungal resistance" OR "anti-malarial resistance" OR "antimalarial resistance" OR zoonosis OR zoonoses OR zoonotic OR "infectious disease" OR "infectious diseases" OR “pandemic prevention”[tiab:~5] OR “pandemic preparedness” [tiab:~5] OR “pandemic response” [tiab:~5] OR “vector disease” [tiab:~5] OR “vector diseases” [tiab:~5] OR "neglected tropical disease" OR "neglected tropical diseases" OR "food safety"

AND

*Governance*

"Organizational Policy"[Mesh] OR "Public Policy"[Mesh] OR governance OR regulatory OR "policy process" OR "policy processes" OR "policy framework" OR "policy frameworks" OR "policy mechanism" OR "policy mechanisms" OR “coordination mechanism” OR “coordination mechanisms”

**Web of Science (Abstract)**

*One Health*

"One Health" OR multisectoral OR multi-sectoral OR cross-sectoral OR intersectoral OR inter-sectoral

AND

*Infectious diseases*

amr OR "antimicrobial resistance" OR abr OR "antibiotic resistance" OR "antimicrobial stewardship" OR "antibiotic stewardship" OR "drug resistance" OR "anti-parasitic resistance" OR "antiparasitic resistance" OR "anti-fungal resistance" OR "antifungal resistance" OR "anti-malarial resistance" OR "antimalarial resistance" OR zoono* OR "infectious disease" OR "infectious diseases" OR pandemic NEAR/5 prevention OR pandemic NEAR/5 preparedness OR pandemic NEAR/5 response OR vector NEAR/5 disease OR vector NEAR/5 diseases OR "neglected tropical disease" OR "neglected tropical diseases" OR "food safety"

AND

*Governance*

governance OR regulatory OR “coordination mechanism” OR “coordination mechanisms” OR "policy process" OR "policy processes" OR "policy framework" OR "policy frameworks" OR "policy mechanism" OR "policy mechanisms"
