# Supplementary material for: Implementing One Health governance approaches to mitigate antimicrobial resistance across institutional, social, economic and political contexts: a scoping review
Source: BMJ Open. 2026 Jul 8;16(7):e115471. doi: 10.1136/bmjopen-2025-115471 (PMC13347904; doi:10.1136/bmjopen-2025-115471)
Supplement: online supplemental file 2 [file bmjopen-16-7-s002.docx]

**Supplementary file 2**

Grey literature search strategy

We conducted searches for grey literature reports using domain-specific Google searches. The search terms used for each organisation were “one health” AND governance AND (“case study” OR “country-specific” OR “country office” OR “mission report”), and the search was limited to PDF files. The first 30 results for each search were screened for inclusion. Where relevant documents were found, additional reference lists were hand-searched for related materials.

The websites of the following organisations were searched:

1. WHO Archives
2. FAO
3. WOAH (formerly OIE)
4. UNEP
5. Euro Health Observatory using the search terms “one health” AND governance
6. EU JAMRAI using search terms “one health” AND governance
7. JPIAMR using search terms “one health” AND governance
8. PAHO using search terms “one health” AND governance
9. Africa CDC using search terms “one health” AND governance
10. AFROHUN using search terms “one health” AND governance

Alternative search terms were tested, such as “policy AND antimicrobial resistance AND implementation” but these yielded results primarily focused on One Health initiative implementation rather than implementation of governance models and mechanisms. The decision to review the first 30 results for each search was taken after piloting the searches and noting that relevance of results was minimal after this threshold.
