# Supplementary material for: Implementing One Health governance approaches to mitigate antimicrobial resistance across institutional, social, economic and political contexts: a scoping review
Source: BMJ Open. 2026 Jul 8;16(7):e115471. doi: 10.1136/bmjopen-2025-115471 (PMC13347904; doi:10.1136/bmjopen-2025-115471)
Supplement: online supplemental file 3 [file bmjopen-16-7-s003.docx]

**Supplementary file 3**

*Initial coding framework to be refined and expanded through inductive coding*

| Theme | Sub-theme |
| --- | --- |
| Wider context | - OH capacity (quality and quantity of trained workforce) - Burden of OH-relevant disease and risk factors - Country income and related resource constraints - Recent and ongoing disruption (e.g., conflict, displaced populations, COVID-19, extreme climate events, economic crises) - Important economic activities (e.g., agricultural production, tourism, primary resource extraction, pharmaceutical production) |
| Existing governance context | Formal dimensions (‘governance as blueprint’)   - Location of political authority (departmental/ministerial areas of responsibility; national vs sub-national areas of responsibility) - Accountability (scrutiny of political activity and accountability relative to different stakeholders such as voters, civil society, industry, regional organisations, and donors/lenders) |
|  | Emergent dimensions (‘governance as reality’)   - Collaborative culture (e.g., history of OH response, power struggles, bureaucratic culture) - Institutional capacity (e.g., political stability, regulatory capacity, corruption, transparency) - Global connectivity (e.g., trading and political relationships, movement of people and goods, alignment with regional or global regulations and norms) |
| One Health governance | Formal dimensions (‘governance as blueprint’)   - Resourcing (e.g., How is One Health governance funded?) - Decision-making (e.g., What is the process by decisions are made?) - Leadership (e.g., Who formally leads One Health governance?) - Participation (e.g., Who has a formal role in One Health governance platforms?) - Coordination (e.g., What are the formal mechanisms for information-sharing across sectors?) - Accountability (e.g., Are there designated accountable bodies for actions agreed on?) - Equity (e.g., Is equity considered in the design of One Health governance?) |
|  | Emergent dimensions (‘governance as reality’)   - Resourcing (e.g., Is resourcing inequitable across sectors?) - Decision-making (e.g., Are there inter-sector conflicts about priorities?) - Leadership (e.g., What role do informal leaders, such as policy champions, play?) - Participation (e.g., What are the attitudes of participants to OH efforts?) - Coordination (e.g., What role do informal relationships play in coordination?) - Accountability (e.g., What role do trusts and relationships play in shaping accountability?) - Adaptiveness (e.g., How does governance adapt in the face of unexpected events?) |
|  | Actors involved (e.g., government ministries, civil society, private sector, research institutions, global and regional organisations, development partners, donors) |
| Performance | Conceptualisation, framing and/or measurement of performance |
|  | Evidence of performance |
|  | Evidence of equity-specific performance (e.g., decreasing or increasing inequities between sectors or populations) |
