# Supplementary figures and images for: Implementing One Health governance approaches to mitigate antimicrobial resistance across institutional, social, economic and political contexts: a scoping review
Source: BMJ Open. 2026 Jul 8;16(7):e115471. doi: 10.1136/bmjopen-2025-115471 (PMC13347904; doi:10.1136/bmjopen-2025-115471)

**Supplementary file 4**


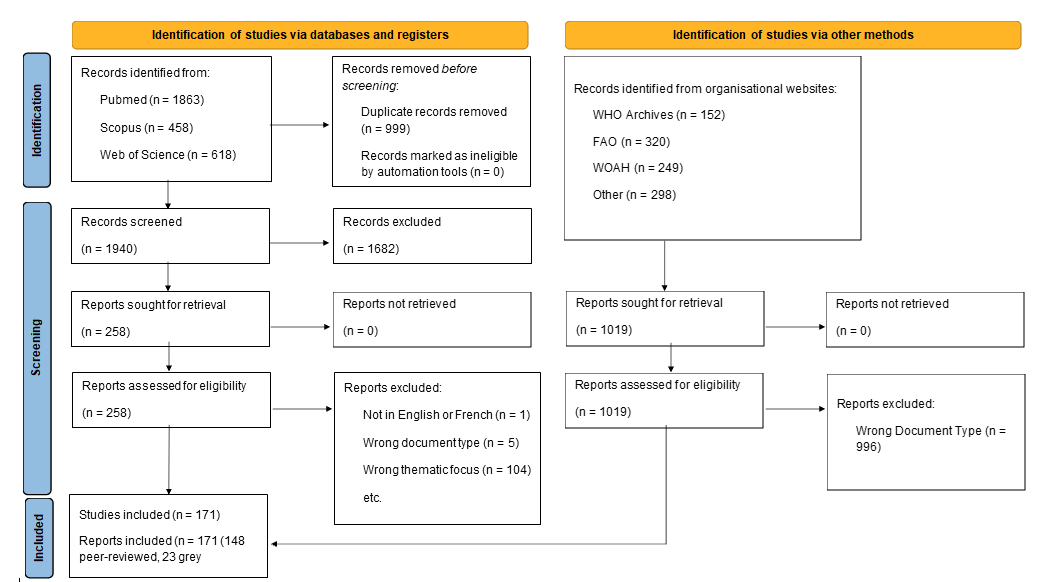


*Figure S1 PRISMA diagram.*

Supplement: online supplemental file 4 [file bmjopen-16-7-s004.docx]
