# Supplementary material for: Implementing One Health governance approaches to mitigate antimicrobial resistance across institutional, social, economic and political contexts: a scoping review
Source: BMJ Open. 2026 Jul 8;16(7):e115471. doi: 10.1136/bmjopen-2025-115471 (PMC13347904; doi:10.1136/bmjopen-2025-115471)
Supplement: online supplemental file 5 [file bmjopen-16-7-s005.docx]

| **Lead author** | **Year** | **Article name** | **Country** | **Region (WB)** | **Income (WB)** | **Data collection period** | **Data sources** | **Aims/Objectives** | **Topic** |
| --- | --- | --- | --- | --- | --- | --- | --- | --- | --- |
| Abass (1) | 2025 | One health in Gulf Cooperation Council countries: A roadmap for integrated human-animal-environment health | Multiple | Middle East and North Africa | N/A | February 2025 | Systematic review | This systematic review aims to compile and analyze One Health-related studies across the six GCC countries, identifying key challenges to implementation, opportunities for strengthening One Health frameworks, barriers, and gaps in adoption, and policy and practice recommendations | One Health |
| Abutarbush  (2) | 2022 | Implementation of One Health approach in Jordan: Review and mapping of ministerial mechanisms of zoonotic disease reporting and control, and inter-sectoral collaboration | Jordan | Middle East and North Africa | Lower-middle | January - June 2021 | Document review; semi-structured interviews | The objective of this study is to review and map the existing structures of ministry of health and ministry of agriculture in relation to zoonotic diseases reporting and control, and inter-sectoral collaboration in Jordan | One Health |
| Abuzerr (3) | 2021 | Implementation challenges of an integrated One Health surveillance system in humanitarian settings: A qualitative study in Palestine | Palestine | Middle East and North Africa | Lower-middle | April -August 2020 | Semi-structured interviews | This study aimed to explore barriers to implementing an integrated OH surveillance system in Palestine | One Health |
| Acharya (4) | 2019 | One health approach in Nepal: Scope, opportunities and challenges | Nepal | South Asia | Lower-middle | Not stated | Not stated | This paper discusses status, challenges and opportunities of OH in Nepal and suggests ways to promote and institutionalize it | One Health |
| Adewumi (5) | 2026 | Intersectoral collaboration for strengthening infectious disease prevention and control in Nigeria: A narrative review | Nigeria | Sub-Saharan Africa | Lower-middle | 2025 | Literature review of empirical research, reviews, and grey literature | This review critically examines intersectoral collaboration for infectious disease prevention and control in Nigeria using Systems Theory to provide unique insights that are consistent with established institutions as well as practical, context-based recommendations based on recent empirical evidence | One Health |
| Adini (6) | 2019 | Earlier detection of public health risks – Health policy lesson for better compliance with the International Health Regulations (IHR 2005): Insights from low-, mid-, and high-income countries | Multiple | N/A | N/A | September 2017 | Workshop | The objective of this paper is to share the insights learned in the workshop concerning earlier detection and facilitate understanding of measures that may support diverse communities in combating public health risks across the three income regions, and reflect on health policy lessons that may be drawn from these insights | Other |
| Adnyana (7) | 2023 | One Health approach and zoonotic diseases in Indonesia: Urgency of implementation and challenges | Indonesia | East Asia and Pacific | Upper-middle | Not stated | Not stated | This review article provides information on the importance of generating research on zoonotic diseases, especially in Indonesia, where research is still relatively scarce | One Health |
| African Centres for Disease Control and Prevention (8) | 2024 | Voicing African priorities on the active pandemic: Accelerating the continental response to antimicrobial resistance | Multiple | Multiple (Middle East and North Africa, Sub-Saharan Africa) | Multiple | N/A | N/A | The comprehensive measures outlined in this report are essential for curbing the threat of AMR in Africa, ensuring the health and prosperity of future generations, and achieving sustainable development goals across the continent | AMR |
| Aggarwal (9) | 2020 | One Health Approach to Address Zoonotic Diseases | India | South Asia | Lower-middle | N/A | N/A | The Government of India has taken some initiatives to tackle burgeoning problems such as antimicrobial resistance, zoonotic diseases, and food safety using the OH approach, but there are several challenges at the level of implementation | One Health |
| Aguiar (10) | 2025 | Equity in the governance of antimicrobial resistance surveillance: Global experts’ perspectives | Multiple | N/A | N/A | March – June 2023 | Semi-structured interviews | We further demonstrate how inclusion of equity as a guiding principle in AMR surveillance can be a framework to re-conceptualize the epidemiological sphere of AMR | AMR |
| Aguiar (11) | 2024 | One health governance of antimicrobial resistance seen through an Urban Political Ecology lens: A critical interpretive synthesis | Multiple | N/A | N/A | March 2022 | Literature search | This article aims to clarify how scientific literature has situated OH-AMR governance responses in relation to six socioecological dimensions: global health threats, broader concerns, governance frameworks, socioeconomic factors, health equity, and environmental justice | AMR |
| Ahmed (12) | 2022 | The Implementation of National Action Plan (NAP) on Antimicrobial Resistance (AMR) in Bangladesh: Challenges and lessons learned from a cross-sectional qualitative study | Bangladesh | South Asia | Lower-middle | January - December 2021 | Rapid literature review, semi-structured interviews | This study was undertaken to explore the current situation of NAP on AMR implementation in Bangladesh and improve understanding of the dynamics of its development, constraints of implementation and perceived measures to overcome these in policy and practice | AMR |
| Allal (13) | 2019 | From Four-Way Linking to a One Health Platform in Egypt: Institutionalisation of a multidisciplinary and multisectoral One Health system | Egypt | Middle East and North Africa | Lower-middle | Not stated | Not stated | The primary purpose of this mechanism is to provide a comprehensive, strategic approach to concurrent and future health challenges that are facing public and animal health, including environmental impacts. | One Health |
| Allel (14) | 2024 | Opportunities and challenges in antimicrobial resistance policy including animal production systems and humans across stakeholders in Argentina: A context and qualitative analysis | Argentina | Latin America and the Caribbean | Upper-middle | September 2022 – February 2023 | Document review; semi-structured interviews | This study aims to better understand the stakeholder and regulatory landscape and the challenges and opportunities Argentina faces in implementing its NAP. | AMR |
| Allen (15) | 2015 | Governance and One Health: Exploring the Impact of Federalism and Bureaucracy on Zoonotic Disease Detection and Reporting | USA | North America | High | Not stated | Document review, semi-structured interviews, survey data | Through a comparative case-study approach, this research explores how federalism, bureaucratic behaviour, and institutional design in the United States may influence zoonotic disease outbreak detection and reporting | One Health |
| Anderson (16) | 2019 | Averting the AMR crisis: What are the avenues for policy action for countries in Europe | Multiple | Europe and Central Asia | Multiple | Not stated | Not stated | The aim of this brief is to present key policy options that can be effective in combating AMR in Europe | AMR |
| Asaaga (17) | 2021 | Operationalising the “One Health” approach in India: facilitators of and barriers to effective cross-sector convergence for zoonoses prevention and control | India | South Asia | Lower-middle | December 2018 - August 2019 | Document review; semi-structured interviews | The study aims to: (1) inform the effective operationalisation of contextually appropriate OH, by improving practical understanding of the policy and local influences on OH implementation, and (2) identify barriers and facilitators linked to the prevention and control of zoonoses | One Health |
| Ayobami (18) | 2021 | COVID-19: an opportunity to re-evaluate the implementation of a One Health approach to tackling emerging infections in Nigeria and other sub-Saharan African countries | Multiple | Sub-Saharan Africa | Multiple | Not stated | Not stated | The aim of this review is to discuss possible strategies for developing an OH policy approach for the SSA health systems, drawing from a critical analysis of the prevailing cross-cutting issues and implementation barriers | One Health |
| Bakiika (19) | 2023 | Contribution of the one health approach to strengthening health security in Uganda: A case study | Uganda | Sub-Saharan Africa | Low | September - October 2020 | Focus groups, document review | This study assessed the contribution of the One Health approach to strengthening health security in Uganda | One Health |
| Barroga (20) | 2018 | Practical inter‐sectoral linking: Tool to rabies One Health coordination to the grass-roots level | Philippines | East Asia and Pacific | Lower-middle | December - June 2017 | Laboratory surveillance data | This study summarizes the development of a OH framework involving multiple sectors, down to the village level in Bicol, Philippines. Linkages amongst animal, human and village officials, along with the operational protocols of each sector have been established. | NTDs |
| Batsukh (21) | 2013 | One Health in Mongolia | Mongolia | East Asia and Pacific | Upper-middle | Not stated | Not stated | In 2011, a list of experts on major zoonoses were identified from different sectors and formed into a taskforce to identify focal points for rabies, bucellosis, and vector-borne diseases. | One Health |
| Belot (22) | 2021 | IHR-PVS National Bridging Workshops, a tool to operationalize the collaboration between human and animal health while advancing sector-specific coals in countries | Multiple | Multiple | Multiple | 2013 - 2017 | Conceptual, workshops | Through a series of six phased pilots, the IHR-PVS National Bridging Workshop method was developed and refined | One Health |
| Beltramo (23) | 2024 | Comparative analysis of One Health policies in Asia for exploring opportunities for British Columbia in Canada | Multiple | Multiple | Multiple | Not stated | Semi-structured interviews, literature review | We conducted a comparative analysis of One Health policies in Asia, specifically Singapore, Hong Kong, Bangladesh, and Thailand, which have well-established and sophisticated One Health approaches, to determine good practices in the implementation of One Health that could be considered for adoption in British Columbia. | One Health |
| Berman (24) | 2023 | Antimicrobial resistance in food-producing animals: towards implementing a one health based national action plan in Israel | Israel | Middle East and North Africa | High | Not stated | Interviews, document review | We review several national action plans against antimicrobial resistance around the world in order to suggest approaches to develop a national action plan in Israel | AMR |
| Beyene (25) | 2023 | Situational analysis of antimicrobial resistance, laboratory capacities, surveillance systems and containment activities in Ethiopia: A new and one health approach | Ethiopia | Sub-Saharan Africa | Low | April - May 2022 | Document review, semi-structured interviews, survey data | This AMR situational analysis was conducted using a new and one health approach to get background information and an overview of the status of AMR on major bacterial pathogens, laboratory capacities, surveillance systems to provide the basis for setting priorities, and develop strategies to mitigate AMR in the country | AMR |
| Bhat (26) | 2021 | Kyasanur Forest Disease, is our surveillance system healthy to prevent a larger outbreak? A mixed-method study, Shivamogga, Karnataka, India: 2019 | India | South Asia | Lower-middle | May 2019 | Document review, semi-structured interviews, survey data | A study was conducted with the following specific objectives: (1) to describe the surveillance system for KFD for humans and animals, (2) to assess the attributes of the human surveillance system against existing guidelines on KFD surveillance in Karnataka, and (3) to identify the issues in the KFD surveillance and prediction mechanisms in the Shivamogga district | One Health |
| Björkman (27) | 2021 | Animal Production With Restrictive Use of Antibiotics to Contain Antimicrobial Resistance in Sweden - A Qualitative Study | Sweden | Europe and Central Asia | High | January - June 2018 | Semi-structured interviews | This qualitative interview study explored perceptions of work to contain ABR among stakeholders in food animal production in Sweden, with focus on broiler production | AMR |
| Bjorkman (28) | 2022 | Swedish Efforts to Contain Antibiotic Resistance in the Environment - A qualitative study among selected stakeholders | Sweden | Europe and Central Asia | High | April - June 2018 | Semi-structured interviews | In this study, we explored perceptions of work to contain antibiotic resistance with a focus on the environment. | AMR |
| Blankart (29) | 2024 | Health literacy, governance and systems leadership contribute to the implementation of the One Health approach: A virtuous circle | N/A | N/A | N/A | N/A | Not Stated | We explore how achieving the Quadripartite Organizations' One Health Joint Plan of Action can be supported by the concepts of 'One Health literacy' and 'One Health governance' and promote both academic and policy dialogue | One Health |
| Bond (30) | 2013 | The evolution of expansion of regional disease surveillance networks and their role in mitigating the threat of infectious disease outbreaks | Multiple | Multiple | Multiple | N/A | Not Stated | We explore how regional disease surveillance networks add value to global disease detection and response by complementing other systems and efforts, by harnessing their power to achieve other goals such as health and human security, and by helping countries adapt to complex challenges via multi-sectoral solutions. | One Health |
| Bordier (31) | 2018 | Antibiotic resistance in Vietnam: moving towards a One Health surveillance system | Vietnam | East Asia and Pacific | Lower-middle | Not stated | Literature review; semi-structured interviews; stakeholder map | To assess the feasibility of operationalising this strategy within the national context, we explored the role of key stakeholders in the strategy, as well as their abilities to comply with it | AMR |
| Bronzwaer (32) | 2024 | The framework for action of the Cross-agency One Health Task Force | Multiple | Europe and Central Asia | High | N/A | N/A | The framework outlines five strategic objectives: enhancing strategic coordination, promoting research, strengthening capacity building, fostering communication and partnerships, as well as joint activities. These objectives will align the agencies’ efforts, improve collaboration, and promote a transdisciplinary approach to address health threats. | One Health |
| Brunskill (33) | 2024 | Seeing the whole elephant: designing ‘one health’ governance to fight antimicrobial resistance | Canada | North America | High | N/A | N/A | As a steering committee charged by the Public Health Agency of Canada with designing effective governance of the national AMR response, our work through 2020 and 2021 gave us a lived experience of this challenge, which involved consulting various stakeholders to reach consensus on functions and structure of a potential Pan-Canadian AMR governance body | AMR |
| Buregyeya (34) | 2020 | Operationalizing the One Health Approach in Uganda: Challenges and Opportunities | Uganda | Sub-Saharan Africa | Low | Not stated | Not stated | In this paper, we present major achievements and challenges of OH implementation, and make recommendations for systematic and sustainable OH implementation | One Health |
| Busani (35) | 2023 | Fighting Antimicrobial Resistance and Healthcare-Associated Infections in EU-JAMRAI: The One-Health Response from Italy | Italy | Europe and Central Asia | High | Not stated | Focus groups, semi-structured interviews | This paper describes the results of EU-JAMRAI relevant to Italy and its impact on national policies | AMR |
| Cediel Becerra  (36) | 2021 | A Survey on One Health Approach in Colombia and Some Latin American Countries: From a fragmented health organization to an integrated health response to global challenges | Multiple (Latin America) | Latin America and the Caribbean | Multiple | 2018-2020 | Survey questionnaire | In order to explore existing collaboration amongst the animal health, human-public health and environmental health sectors, and describe the perception and knowledge on OH in Columbia and other countries in Latin America, a questionnaire-based survey was circulated amongst main stakeholders | One Health |
| Cham (37) | 2024 | Can global health security frameworks measure One Health implementation in West Africa? A mixed-methods study | West Africa (ECOWAS countries) | Sub-Saharan Africa | Multiple | March - April 2022 | Semi-structured interviews, external indicator (e.g., IHR data) | This study sought to assess and explore whether the existing metrics of global health security frameworks can measure the successful implementation of OH activities, evaluate the progress made since 2016, and identify key areas for improvement in the region | One Health |
| Chanda (38) | 2008 | Integrated vector management: The Zambian experience | Zambia | Sub-Saharan Africa | Lower-middle | 2003 - 2007 | the Integrated vector management process in Zambia | This paper reports on the IVM processes, achievements and the status of key elements of IVM over the past five years | Other |
| Chatterjee (39) | 2016 | Integrating one health in national health policies of developing countries: India's lost opportunities | India | South Asia | Lower-middle | Not stated | Not stated | The adoption of One Health approaches in health and related sectoral policies is a critical policy requirement for India and other developing countries | One Health |
| Chattopadhyay (40) | 2018 | A Qualitative Stakeholder Analysis of Avian Influenza Policy in Bangladesh | Bangladesh | South Asia | Lower-middle | February - May 2016 | Semi-structured interviews, focus group | This study specifically aimed to identify the future policy options to prevent and control avian influenza and other poultry-related zoonotic diseases in Bangladesh | Avian Influenza |
| Chen (41) | 2026 | Evaluation of antimicrobial resistance governance across 193 countries to inform the 2026 Global Action Plan update | Multiple | Multiple | Multiple | 2017 – 2022 | Multidimensional One Health governance index and structured Delphi consultation | By integrating multidimensional governance indicators with longitudinal AMR outcome data, this study quantitatively assesses progress in national AMR governance across policy design, implementation and monitoring. It further examines whether national AMR governance has contributed to measurable improvements in AMR-related outcomes since the adoption of the GAP. | AMR |
| Cheptoyek (42) | 2024 | Utilization and associated determinants of multi-sectoral approach in zoonotic disease surveillance among animal and human healthcare workers in Nakuru County, Kenya | Kenya | Sub-Saharan Africa | Lower-middle | August – October 2023 | Semi-structured interviewer-administered questionnaire | The aim of this study was to determine the level of utilization of MZDS (multi-sectoral collaboration in zoonotic disease surveillance) and its associated factors among animal and human healthcare workers in Nakuru county, Kenya | One Health |
| Chongo (43) | 2024 | Outcomes from a zoonotic disease prioritization workshop using One Health approach in Mozambique, 2018 to 2023 | Mozambique | Sub-Saharan Africa | Low | 2018 - 2023 | Workshops | The current manuscript describes the process and importance of the zoonotic disease prioritization carried out in Mozambique, and how the prioritization of zoonotic diseases has supported advancing One Health after the OHZDP workshop | One Health |
| Chua (44) | 2021 | An analysis of national action plans on antimicrobial resistance in Southeast Asia using a governance framework approach | Multiple | Multiple (South Asia, East Asia and Pacific) | Multiple | Not stated | Ten National Action Plans | As the validity periods of most NAPs are ending, an analysis now will provide an opportunity to improve subsequent iterations of these NAPs. We analysed the current NAPs of ten ASEAN countries. We explored their objective alignment with GAP and performed content analysis using an AMR governance framework | AMR |
| Chua (45) | 2023 | A Qualitative Study on the Policy Process and Development of the National Action Plan on Antimicrobial Resistance in Singapore | Singapore | East Asia and Pacific | High | November 2020 - October 2021 | Semi-structured interviews | In this study, we analysed these aspects (the policy process and development of the NSAP in Singapore) using an AMR governance framework | AMR |
| Chunsuttiwat (46) | 2008 | Response to avian influenza and preparedness for pandemic influenza: Thailand's experience | Thailand | East Asia and Pacific | Upper-middle | N/A | N/A | Thailand is joining global efforts in pandemic influenza preparedness. The national preparedness plan highlights building of national capacity for self-reliance and regional/international cooperation | Avian Influenza |
| Collineau (47) | 2024 | Moving towards One Health surveillance of antibiotic resistance in France: A semi-quantitative evaluation of the level of collaboration within the national surveillance systems | France | Europe and Central Asia | High | March - May 2021 | Semi-structured interviews, literature review | This study aimed to evaluate collaboration within this system and to formulate recommendations towards more integration | AMR |
| Corrêa (48) | 2023 | The governance of antimicrobial resistance in Brazil: Challenges for developing and implementing a one health agenda | Brazil | Latin America and the Caribbean | Upper-middle | June - December 2021 | Semi-structured interviews | This article explores stakeholders' perceptions of the challenges for developing a OH agenda to tackle AMR in Brazil, including the development and implementation of the Brazilian National Action Plan | AMR |
| Davido (49) | 2026 | Strengthening antimicrobial resistance governance in Europe: A coordinated one health approach | Multiple | Europe and Central Asia | Multiple | Not Stated | Analysis of policy gaps and operational barriers | By analyzing policy gaps and operational barriers, this paper underscores the need for stronger accountability and political commitment to translate strategies into sustainable action. | AMR |
| Dayapera (50) | 2024 | One health in the Philippines: A review and situational analysis | Philippines | East Asia and Pacific | Lower-middle | July - August 2023 | Semi-structured interviews, document review, workshops | This paper examines these issues (complex human, animal, and environmental health issues), existing interventions and their implementation challenges | One Health |
| de Best (51) | 2025 | One Health preparedness and response for mosquito-borne viruses: A stakeholder-and social network-analysis in the Netherlands | Netherlands | Europe and Central Asia | High | 2021 - 2023 | Semi-structured interviews | This study aims to identify stakeholders in the field of preparedness and response to mosquito-borne viruses (MBVs) in the Netherlands and map collaborations, knowledge- and information-sharing between these stakeholders, their domains, and governance levels. In addition, we aim to identify bottlenecks in these networks and uncover underlying reasons | One Health |
| De La Rocque (52) | 2023 | One health systems strengthening in countries: Tripartite tools and approaches at the human-animal-environment interface | Multiple | Multiple | Multiple | Not stated | Conceptual, workshops | This paper highlights the historical background of this collaboration in the specific area of health security, using country examples to demonstrate lessons learnt and the evolution and pairing of Tripartite programs and processes to jointly develop and deliver capacity strengthening tools to countries and strengthen performance for iterative evaluations | One Health |
| Degeling (53) | 2015 | Implementing a One Health approach to emerging infectious disease: reflections on the socio-political, ethical and legal dimensions | Multiple | Multiple | Multiple | Not stated | International / government reports, academic databases, news, organizational newsfeeds, and websites of major OH collaborations | We draw on this data to provide guidance as to how these concerns and issues might be addressed, and point to remaining challenges to the likely success of the One Health approach to EID control and prevention | One Health |
| Delpy (54) | 2024 | Integrated surveillance systems for antibiotic resistance in a One Health context: A scoping review | Multiple | Multiple | Multiple | January 2000 - January 2022 | primary and secondary peer-reviewed literature in French and English | A scoping review that analyses: (i) the organizational and functional characteristics of existing integrated surveillance systems for ABR; (ii) the socio-economic and political context in which they operate; and (iii) the levels of integration reached in these systems and their related outcomes. | AMR |
| Denis-Robichaud (55) | 2024 | One Health communication channels: a qualitative case study of swine influenza in Canada in 2020 | Canada | North America | High | November - December 2021 | Semi-structured interviews, document analysis | The objectives of this qualitative case study were to describe the communication channels used between human and animal health stakeholders and to identify the elements that have enabled the integration of the One Health approach | One Health |
| Deruelle (56) | 2021 | A tributed to the foot soldiers: European health agencies in the fight against antimicrobial resistance | Multiple | Europe and Central Asia | Multiple | Not stated | Textual sources of agencies, in-depth interviews | The aim of this paper is twofold: first as a tribute to the 'foot soldiers,' it details the scientific cooperation of agencies which defined the action taken at EU level to fight AMR. Second, this paper addresses the following question: how are tasks and roles ultimately assigned in inter-agency cooperation on AMR? | AMR |
| Don Bamunusinghage Nihal (57) | 2020 | Challenges and opportunities for wildlife disease surveillance in Sri Lanka | Sri Lanka | South Asia | Lower-middle | August - November 2015 | Survey questionnaire | This study examined perceived obstacles and opportunities to undertake wildlife disease surveillance in Sri Lanka | One Health |
| El-Jardali (58) | 2024 | Multi-sectoral collaborations in selected countries of the Eastern Mediterranean region: Assessment, enablers and missed opportunities from the COVID-19 pandemic response | Multiple | Multiple (Middle East and North Africa, Sub-Saharan Africa) | Multiple (High-income; Lower-Middle income; low-income) | Not stated | Comprehensive document and key informant interviews | This study aims to generate evidence on the extent to which multi-sectoral collaborations have been employed in the macro-level responses to the COVID-19 pandemic in nine selected countries of the Eastern Mediterranean region. | One Health |
| Erkyihun (59) | 2022 | A review on One Health approach in Ethiopia | Ethiopia | Sub-Saharan Africa | Low | Not stated | Published literature; data publicly available on WHO, FAO, US CDC, ILRI, Ethiopia's One Health ministries’ websites; consultations with stakeholders | This review is believed to highlight potential areas of collaboration between the Ethiopian medical, veterinary sector and other scientific communities | One Health |
| Espeschit (60) | 2021 | Public Policies and One Health in Brazil: The Challenge of the Disarticulation | Brazil | Latin America and the Caribbean | Upper-middle | N/A | N/A | This perspective aims to describe the government instruments that constitute potential national efforts and the challenges for the consolidation of the One Health initiative in Brazil | One Health |
| European Union (61) | 2022 | Member states' one health national action plans against antimicrobial resistance | Multiple | Europe and Central Asia | Multiple | 2021 | Information submitted by European Member States re NAPs | This report summarises a review of European Union Member States' One Health AMR NAPs as of 1 September 2021. | AMR |
| FAO (62) | 2008 | Contributing to one world, one health: A strategic framework for reducing risks of infectious diseases at the animal-human-ecosystems interface | Multiple | Multiple | Multiple | N/A | N/A | The objectives and outputs of the Strategic Framework focus on some of the major drivers for emergence, spread and persistence of EID. | One Health |
| FAO (63) | 2011 | One Health: Seeing around corners. A regional communication strategy framework against infectious diseases in Asia and the Pacific 2011-2016 | Multiple | Multiple (East Asia and Pacific, South Asia) | Multiple (Upper-Middle; Lower-Middle) | 2003 - 2010 | Two consultations and a literature review | This document takes FAO's emerging vision a step forward, and reflects the need to root future communication in prevention strategies in addition to emergency response, basing interventions on community ownership and engagement, understanding that changes in behaviour and practices will arise from appreciating the long-term benefits in protecting livelihoods and health that accrue from mitigating. the emergence of new diseases or recurrence of old ones | One Health |
| FAO (64) | 2011 | 4th Report: Global Programme for the prevention and control of highly pathogenic avian influenza | Multiple | Multiple | Multiple | January - December 2010 | Qualitative assessments within FAO Global Programme | A qualitative assessment and review of programmes and country capacities was carried out in five key countries which are at the centre of the agro-ecological zones where H5N1 HPAI is endemic. The countries were assessed in terms of (i) FAO's contribution to the national HPAI response in 2010; (ii) progress with developments of country capacity over the past 12 months; and (iii) observations on the impact of FAO's contribution in the country and the region | Avian Influenza |
| FAO (65) | 2013 | 5th Report: Global programme for the prevention and control of highly pathogenic avian influenza | Multiple | Multiple | Multiple | January 2011 - January 2012 | HPAI global programme activities | This 5th report on the FAO global programme on HPAI covers the period 2011-2012 and provides an overview of the disease situation, the activities conducted and the strategic approach with respect to the reduction of infection in endemically infected countries. | Avian Influenza |
| FAO/OIE/WHO Joint Scientific Consultation Writing Committee (66) | 2011 | Influenza and other emerging zoonotic diseases at the human-animal interface | Multiple | Multiple | Multiple | N/A | N/A | This Tripartite Concept Note focused on building on the ideas from joint Technical meetings focused on pandemic and avian influenza and emerging viral zoonoses and attempting to identify the commonalities among diseases, to provide a scientific basis for collaborative, multisectoral actions. |  |
| FAO/UNEP/WHO/WOAH (67) | 2023 | A guide to implementing the One Health Joint Plan of Action at national level | Multiple | Multiple | Multiple | N/A | N/A | this guide was developed through a participatory process, led by the Quadripartite organizations involving staff engaged in One Health across the headquarters and regional levels of the four organizations. The actions presented in the OH JPA and this accompanying guide aim to inspire greater and more targeted One Health action at every level, enhancing capacities and capabilities to prevent and mitigate risks and threats | One Health |
| FAO/WOAH/WHO (68) | 2019 | Taking a multisectoral, one health approach: A tripartite guide to addressing zoonotic diseases in countries | Multiple | Multiple | Multiple | N/A | N/A | The purpose of this Tripartite Zoonoses Guide is to provide countries with operational guidance and tools for the implementation of a multisectoral, One Health approach to address zoonotic diseases and other shared health threats at the human-animal-environment | One Health |
| Farag (69) | 2019 | Survey on implementation of one health approach for MERS-CoV preparedness in Gulf Cooperation Council and Middle East Countries | Multiple | Middle East and North Africa | Multiple | Not stated | FAO, OIE, WHO and CDC documents; meeting reports; and policy documentation; Questionnaire | To gauge a preliminary understanding about the extent to which the involved countries were using a One Health approach and how it was translated in government policies and practices, the OH working group conducted a survey before the workshop. | One Health |
| Feng (70) | 2024 | Advancing knowledge of One Health in China: lessons for One Health from China's dengue control and prevention programs | China | East Asia and Pacific | Upper-middle | 2024 | Literature review, semi-structured interviews | The review aims to: (1) systematically analyze lessons from China's dengue control and prevention programs, focusing on the integration of these efforts with the OH approach; (2) underscore the reasons of optimizing the dengue control and prevention program; (3) highlight the alignment of China's dengue control strategies with the OH framework; (4) contribute to global efforts in combating dengue, providing scientific evidence and strategic recommendations for other regions facing similar challenges | NTDs |
| Fotakis (71) | 2024 | Leishmaniasis in Greece: Prospects of transitioning to a One Health surveillance system | Greece | Europe and Central Asia | High | 2022 - 2023 | Literature reviews, semi-structured interviews, survey | We aimed to provide an overview of the state of leishmaniasis surveillance in Greece, investigating the prospect of transitioning to a OH surveillance system. | NTDs |
| Frumence (72) | 2021 | The Governance and Implementation of the National Action Plan on Antimicrobial Resistance in Tanzania: A qualitative study | Tanzania | Sub-Saharan Africa | Lower-middle | Not stated | Semi-structured interviews | We aimed to analyze the implementation of the NAP on AMR in Tanzania using the governance framework | AMR |
| Frumence (73) | 2021 | Policy actors and human and animal health practitioners’ perceptions of antimicrobial use and resistance in Tanzania: A qualitative study | Tanzania | Sub-Saharan Africa | Lower-middle | June 2019 - February 2020 | Semi-structured interviews | To explore and describe the perceptions of policy actors and practitioners on antimicrobial use and resistance in human and animal health in Tanzania | AMR |
| Gao (74) | 2025 | The application of One Health concept in China and its practice and innovation in cross-sector cooperation | China | East Asia and Pacific | Upper-Middle | June 2023 – August 2023 | Semi-structured interviews | The purpose of this study is to document and summarize major achievements and identify policy and practice gaps through interviews with various departments of the Chinese government to support the development of One Health in China and worldwide | One Health |
| Gautier (75) | 2023 | The emergence of the Biodiversity/Health nexus: Making biodiversity a health issue | France | Europe and Central Asia | High | Not Applicable | Mixed | We will grasp the structural contradictions and even the denied intentions of this announced policy of "biodiverse health," by taking the option of grasping the mechanisms of the trajectory of the Biodiversity/Health nexus onto the agenda in French political and administrative life, the methods used to do so, and its governance implications linked to the One Health agenda. | One Health |
| Gebrekidan (76) | 2024 | Impact of improper municipal solid waste management on fostering One Health approach in Ethiopia - challenges and opportunities: A systematic review | Ethiopia | Sub-Saharan Africa | Low | Not stated | Literature review, document review | This systematic review examines the impact of improper municipal solid waste on fostering One Health approaches at the national level of Ethiopia by identifying key challenges and opportunities | One Health |
| Glover (77) | 2023 | The WHO costing and budgeting tool for national action plans on antimicrobial resistance - a practical addition to the WHOle toolkit | Multiple | Multiple | Multiple | N/A | N/A | In this brief report we review this WHO costing and budgeting tool, discuss the strengths and weaknesses, and consider its place alongside other health economics and policy-support tools developed | AMR |
| Gobena (FAO) (78) | 2024 | Regulatory frameworks to address antimicrobial resistance in the food and agriculture sectors | Multiple | Multiple | Multiple | N/A | N/A | This study is designed to demonstrate the various ways in which key AMR risks and challenges can be tackled through legislation | AMR |
| Goryoka (79) | 2021 | Prioritizing zoonotic diseases using a multisectoral, One Health approach for The Economic Community of West African States (ACOWAS) | Multiple | Sub-Saharan Africa | Multiple | 2018 | Workshops, qualitative, semi-quantitative, quantitative methods | This publication describes the OHZDP Process and outcomes of the ECOWAS OHZDP Workshop and next steps needed to advance health security in West Africa and beyond | One Health |
| Grant (80) | 2015 | Stakeholder Narratives on Trypanosomiasis, Their Effect on Policy and the Scope for One Health | Zambia | Sub-Saharan Africa | Lower-middle | July - October 2013 | Semi-structured interviews | This paper focuses on exploring the following: The narratives taken by different actors in the field; Why different narratives have become dominant at different points in history; the power relations of stakeholders | NTDs |
| Griffiths (81) | 2013 | Public health responses to a dengue outbreak in a fragile state: A case study of Nepal | Nepal | South Asia | Lower-middle | Not stated | In-depth interviews and focus group discussions | This paper evaluates the dengue case notification, surveillance, laboratory facilities, intersectoral collaboration, and how government and community services responded to the outbreak | NTDs |
| Halabi (82) | 2020 | Adaptation of animal and human health surveillance systems for vector-borne diseases accompanying climate change | USA | North America | High | Not Stated | Not Stated | This article assesses the adverse effects of this splintered framework for detecting and responding to vector-borne infectious diseases currently, how those adverse effects are likely to be manipulated by climate change, and recommends changes in federal law to address them (specifically law governing relevant agencies) | One Health |
| Häsler (83) | 2020 | Reflecting on One Health in Action During the COVID-19 Response | Multiple (Australia, Ireland, Sub-Saharan Africa) | Multiple (East Asia and pacific, Europe and Central Asia, Sub-Saharan Africa) | Multiple | 2020 | Authors' experiences | Here we describe three case studies from state (New South Wales, Australia), national (Ireland), and international (sub-Saharan Africa) scales which illustrate different aspects of One Health in action in response to the COVID-19 pandemic | One Health |
| Hassan-Kadle (84) | 2024 | One health in Somalia: Present status, opportunities and challenges | Somalia | Sub-Saharan Africa | Low | Not stated | Not stated | The present status, opportunities, and challenges of OH in Somalia are discussed in this paper, along with recommendations on institutionalizing and promoting OH in action in the country | One Health |
| Hegewisch-Taylor (85) | 2024 | Analyzing One Health governance and implementation challenges in Mexico | Mexico | Latin America and the Caribbean | Upper-middle | October 2020-April 2021 | Document review, semi-structured interviews | The present study aims to (1) provide a first assessment of the status of the OH governance elements and (2) analyze the enablers, barriers, and recommendations in Mexico to institutionalise a successful OH governance | One Health |
| Hein (86) | 2022 | Fighting Antimicrobial Resistance: Development and Implementation of the Ghanaian National Action Plan (2017-2021) | Ghana | Sub-Saharan Africa | Lower-middle | February -March 2021 | Key informant interview, literature review | In this paper, we present a qualitative analysis of the development of AMR-related policies in Ghana, including the NAP | AMR |
| Hoque (87) | 2020 | Tackling antimicrobial resistance in Bangladesh: A scoping review of policy and practice in human, animal and environment sectors | Bangladesh | South Asia | Lower-middle | August 2019 | Literature review, document review | This review presents a 'snap shot' of the current situation including existing policies and practices to address AMR, and the challenges and barriers associated with their implementation | AMR |
| Humboldt-Dachroeden (88) | 2023 | Translating One Health Knowledge Across Different Institutional and Political Contexts in Europe | Multiple | Europe and Central Asia | Multiple | March - July 2021 | Online Survey (mixed methods) of experts | The aim of this study is to comprehend institutional and political structures that enable the knowledge translation process for the One Health approach. | One Health |
| Igihozo (89) | 2022 | An environmental scan of one health preparedness and response: The case of the COVID-19 pandemic response in Rwanda | Rwanda | Sub-Saharan Africa | Low | August - December 2020 | Published and grey literature | Our objective was to conduct an environmental scan to examine Rwanda's OH structure and its role in the country's response to COVID-19 | One Health |
| Innes (90) | 2022 | Enhancing global health security in Thailand: Strengths and challenges of initiating a One Health approach to avian influenza surveillance | Thailand | East Asia and Pacific | Upper-middle | Not stated | In-depth key informant interviews; Thailand's AI surveillance system | This research aims to describe the AI surveillance system's structure, map the network of stakeholders involved, and illustrate the strengths and challenges of the system's activities. | Avian Influenza |
| Interagency coordination group on antimicrobial resistance (91) | 2019 | No Time to Wait: Securing the future from drug-resistant infections | Multiple | Multiple | Multiple | Not Applicable | Not Applicable | This report presents the IACG's response to the request from Member States in the 2016 political declaration and makes recommendations for urgent action for consideration by the Secretary-General, Member States and other stakeholders in the global response to antimicrobial resistance | AMR |
| Iwu (92) | 2021 | An insight into the implementation of the Global Action Plan on Antimicrobial Resistance in the WHO African Region: A Roadmap for Action | Multiple | Multiple | Multiple | 2018 - 2019 | Open access WHO/FAO/OIE database for GAP implementation | This study evaluated the current status of implementation of the GAP on AMR in WHO African countries via a retrospective, cross-sectional analysis of routinely collected data on AMR | AMR |
| Johnson (93) | 2017 | The challenges of implementing an integrated One Health surveillance system in Australia | Australia | East Asia and Pacific | High | August - September 2016 | Semi-structured interviews | This study aims to explore professionals' perceptions on the challenges of implementing an integrated national One Health surveillance system in Australia | One Health |
| Joshi (94) | 2021 | Strengthening multisectoral coordination on antimicrobial resistance: A landscape analysis of efforts in 11 countries | Multiple | Multiple (South Asia, Sub-Saharan Africa) | Multiple | 2018 - 2020 | JEE | This paper describes the technical approach used by a donor-funded program to strengthen multisectoral coordination on AMR in 11 countries as part of their efforts to advance the objectives of the Global Health Security Agenda and discusses some of the challenges and lessons learned | AMR |
| Khan (95) | 2020 | What are the barriers to implementing national antimicrobial resistance action plans? A novel mixed-methods policy analysis in Pakistan | Pakistan | South Asia | Lower-middle | Not stated | Interviews; a novel card game-based methodology to investigate policy actors' support for implementation | Our study systematically identifies policy actors that influence implementation of action plans on inappropriate use of antibiotics in humans and animals in one high AMR burden LMIC - Pakistan - and investigate the extent to which the most influential policy actors would support implementation of different regulatory approaches | AMU |
| Kimani (96) | 2019 | Expanding beyond zoonoses: The benefits of a national One Health coordination mechanism to address antimicrobial resistance and other shared health threats at the human-animal-environment interface in Kenya | Kenya | Sub-Saharan Africa | Lower-middle | Not stated | Key informant interviews | This paper summarises a series of interviews (with respondents and key informants) that describe how AMR institutionalisation evolved in Kenya. | AMR |
| Koduah (97) | 2021 | Antimicrobial resistance national level dialogue and action in Ghana: Setting and sustaining the agenda and outcomes | Ghana | Sub-Saharan Africa | Lower-middle | May 2019-March 2020 | Semi-structured interviews, document analysis | To examine the processes of setting and sustaining AMR issues on government agenda, the policy actors involved and resulting outcomes | AMR |
| Kolla (98) | 2022 | Enhancing inter-organizational collaboration for wildlife disease surveillance in Sri Lanka | Sri Lanka | South Asia | Lower-middle | July 2015 - August. 2016 | Interviews | Our specific research objective was to conduct a mixed-method study that: (1) determine the existing communication channels and perceived adequacy of communication regarding wildlife, domestic and livestock disease surveillance within & between the participants of the SLWHC. (2) Characterized self-identified gaps in communication within and between the participants of the SLWHC. (3) Identified best practices that can help SLWHC clarify and secure participants' common interests | One Health |
| Lâm (99) | 2024 | How do food safety technical working groups within a One Health framework work? Experiences from Vietnam and Ethiopia | Multiple (Vietnam, Ethiopia) | Multiple (East Asia and Pacific, Sub-Saharan Africa) | Multiple (Lower-middle income; Low-income) | Not stated | Meeting minutes, relevant publications on websites of FSTWGs; authorship team expertise | The aim of this study is to systematically document the development process of established groups in Vietnam and Ethiopia. | One Health |
| Li (100) | 2021 | Wild animal and zoonotic disease risk management and regulation in China: Examining gaps and One Health opportunities in scope, mandates, and monitoring systems | China | East Asia and Pacific | Upper-middle | Not stated | Current laws and regulations, government reports & policy documents, existing literature on zoonotic disease preparedness and prevention | This article primarily focuses on the systems in place to regulate at-risk human-animal interactions in wild animal trade and captive-breeding relevant to forestry, agriculture, and public health sectors | One Health |
| Lin (101) | 2017 | Progressing the sustainable development goals through health in All Policies: Case studies from around the world | Multiple | Multiple | Multiple | N/A | N/A | This book aims to contribute to an improved knowledge base as well as to enhanced public health practice in particular by focusing on HiAP examples at different levels of development and maturity | One Health |
| Lioupi (102) | 2025 | Harnessing medical bioethics mediation to advance One Health governance | Multiple | N/A | N/A | N/A | Conceptual review | This paper develops a conceptual framework for integrating medical bioethics mediation into OH governance and illustrates its relevance through examples from zoonotic disease control, AMR, and environmental health. The aim is to demonstrate how mediation’s core principles, neutrality, confidentiality, respect, and structured dialogue, can strengthen trust, ethical deliberation, and multisectoral cooperation within OH systems | One Health |
| Lokossou (103) | 2021 | Operationalizing the ECOWAS regional one health coordination mechanism (2016-2019): Scoping review on progress, challenges, and way forward | Multiple | Sub-Saharan Africa | Multiple | 2016 - 2019 | Scoping review; desk review; interview of key informants; viewpoints of relevant stakeholders | The purpose of this paper is to document and summarize the key achievements and identify the gaps in policy and practice to support the development of R-OHCM in West Africa | One Health |
| Lota (104) | 2022 | A qualitative study on the design and implementation of the national action plan on antimicrobial resistance in the Philippines | Philippines | East Asia and Pacific | Lower-middle | October 2020 - May 2021 | In-depth interviews | Our study aims to discuss the policy process of developing and implementing the NAP on AMR via in-depth interviews to identify policy lessons and the best practices to guide advocacy regarding AMR policy | AMR |
| Lowe (105) | 2020 | Building resilience to mosquito-borne diseases in the Caribbean | Caribbean | Multiple | Multiple | Not stated | N/A | We discuss progress towards bridging the gap between climate science and public health decision-making in the Caribbean to build health system resilience to extreme climatic events. | One Health |
| McKenzie (106) | 2016 | One health research and training and government support for one health in South Asia | Multiple | South Asia | Multiple (upper-middle income; lower-middle income; low-income income) | 1996 - 2016 | Peer-reviewed publications | The objectives of this article are to describe OH research and training and capacity building activities and the important developments in government support for OH in these countries to identify current achievements and gaps | One Health |
| McPake (107) | 2022 | Role of regulatory capacity in the animal and human health systems in driving response to zoonotic disease outbreaks in the Mekong region | Multiple (Cambodia, Laos, Vietnam) | East Asia and Pacific | Lower-middle | Not Stated | Regulatory policies/capacities | This paper seeks to explore different regulatory capacities in the animal and human health systems, and how these impact zoonotic disease responses using Avian Influenza (AI) in the Mekong as a case study | Avian Influenza |
| McPherson (108) | 2018 | Exploring governance for a One Health collaboration for leptospirosis prevention and control in Fiji: Stakeholder perceptions, evidence and processes | Fiji | East Asia and Pacific | Upper-middle | 2014 | Semi-structured interviews | This paper presents the findings of qualitative research to inform policy around governance for a One Health multisectoral approach to leptospirosis control | NTDs |
| Mhone (109) | 2025 | One Health: Governance and regulatory framework for antimicrobial use in Malawi | Malawi | Sub-Saharan Africa | Low | May 2023 – June 2023 | Document review, Semi-structured interviews, Governance mapping | This study aimed to analyze the governance and regulatory framework for AMU in Malawi’s agricultural sector, identify existing gaps, and provide recommendations for strengthening AMU policies. | AMR |
| Mickelsson (110) | 2025 | Agile policies for antimicrobial resistance: A contextual approach to sustainable health challenges | Zimbabwe | Sub-Saharan Africa | Lower-middle | Not stated | Participatory research workshops | The aim of this paper is to explore contextual conditions for AMR policy implementation in Zimbabwe | AMR |
| Mitchell (111) | 2020 | The challenges of investigating antimicrobial resistance in Vietnam - what benefits does a One Health approach offer the animal and human health sectors | Vietnam | East Asia and Pacific | Lower-middle | August 2018 - February 2019 | Semi-structured interviews | To advance understanding of the willingness and abilities of the human and animal health sectors to undertake investigations of AMR with a One Health approach, we explored the perceptions and experiences of those tasked with investigating AMR in Vietnam, and the benefits a multi-sectorial approach offers | AMR |
| Molina-Flores (112) | 2025 | Assessment of One Health initiatives from a veterinary public health approach in Latin America and the Caribbean | Multiple | Latin America and the Caribbean | Multiple | July 2022 | Workshop and document review | This study aims to develop a baseline for Latin America and the Caribbean, focusing on One Health intersectoral actions. | One Health |
| Mor (113) | 2023 | Organising for One Health in a developing country | Multiple (India, Bangladesh, Kenya, Rwanda) | Multiple (South Asia, Sub-Saharan Africa) | Multiple (Lower-middle income; low-income) | Not Applicable | Not Applicable | The paper analyses the success and failures associated with the way in which India, Bangladesh, Kenya, and Rwanda have organised for One Health. | One Health |
| Moura (114) | 2023 | Evaluating the OH-EpiCap tool using the Danish integrated surveillance program for AMU and AMR as a case study | Denmark | Europe and Central Asia | High | 2022 | OH-EpiCap and DANMAP | We aimed to evaluate DANMAP using OH-EpiCap and hereby assessed the suitability of OH-EpiCap to evaluate integrated AMR surveillance systems | AMR |
| Mphande-Nyasulu (115) | 2024 | Outbreak preparedness and response strategies in ASEAN member states: A scoping review | Multiple | East Asia and Pacific | Multiple | 2001 - 2022 | Scoping review | This review aimed to compile and analyze outbreak preparedness and response strategies, highlighting the success of coordinated multi-sectoral approaches and policy responses within the ASEAN region | One Health |
| Mulenga (116) | 2021 | Policy and Linkages in the Application of a One Health System for Reporting and Controlling African Trypanosomiasis and Other Zoonotic Diseases in Zambia | Zambia | Sub-Saharan Africa | Lower-middle | Not stated | Document review; literature review; survey | This study explored the impact of the Zambian government policies on animal and human disease reporting and management and on One Health opportunities. | NTDs |
| Munyua (117) | 2019 | Successes and challenges of the One Health approach in Kenya over the last decade | Kenya | Sub-Saharan Africa | Lower-middle | N/A | N/A | Here, we highlight the successes and challenges of the GDDD (now called the Division of Global Health Protection) in the last decade, specifically in 1) developing institutional capacity for OH implementation, 2) strengthening capacity for surveillance and reporting in animal health sector and 3) expanding the research capacity in Kenya and the East Africa region. | One Health |
| Mwacalimba (118) | 2015 | ‘One health’ and development priorities in resource-constrained countries: Policy lessons from avian and pandemic influenza preparedness in Zambia | Zambia | Sub-Saharan Africa | Lower-middle | June - December 2009 | Semi-structured interviews, document review, participant observation | This article draws on a policy study of national level avian and pandemic influenza preparedness between 2005 and 2009 across the sectors of trade, health and agriculture in Zambia | Avian Influenza |
| Nair (119) | 2021 | India’s National Action Plan on Antimicrobial Resistance: a critical perspective | India | South Asia | Lower-middle | N/A | N/A | In this paper, we examine the implementation, scope, and progress of the NAP-AMR in India with respect to the human sector | AMR |
| Nana (120) | 2022 | Towards an integrated surveillance of zoonotic diseases in Burkina Faso: The case of anthrax | Burkina Faso | Sub-Saharan Africa | Low | March - October 2021 | Semi-structured interview, literature review | Based on stakeholder perspectives, the study has for objective to deepen our understanding of the anthrax surveillance system and to identify the obstacles and levers towards a more integrated approach to anthrax surveillance in Burkina Faso. | Other |
| Nana (121) | 2024 | A participatory approach to move towards a One Health surveillance system for anthrax in Burkina Faso | Burkina Faso | Sub-Saharan Africa | Low | September 2022 | Workshop | A participatory workshop was held with representatives of the different categories of surveillance stakeholders to collectively define such a shared vision, and then identify the changes, and associated actions, necessary to evolve from the current situation to the desired situation | Other |
| Nantima (122) | 2019 | The importance of a One Health approach for prioritising zoonotic diseases to focus on capacity-building efforts in Uganda | Uganda | Sub-Saharan Africa | Low | N/A | N/A | The purpose of this paper is to: (a) highlight the importance of using a OH approach for prioritising zoonotic diseases in Uganda, thereby moving away from sector-specific activities and plans; (b) highlight the advances made in capacity building based on experience from the most recent zoonotic disease outbreak responses and the implementation of Uganda's NOHP and National OH Strategic Plan | One Health |
| Nguendo-Yongsi (123) | 2022 | Intersectoral collaboration for healthier human settlements: perceptions and experiences from stakeholders in Douala, Cameroon | Cameroon | Sub-Saharan Africa | Lower-middle | Not stated | Semi-structured interviews | This study explored stakeholder perspectives on health and housing challenges in Douala, Cameroon, as well as their experiences of intersectoral collaboration to address these challenges | One Health |
| Nguyen (124) | 2019 | Progress towards rabies control and elimination in Vietnam | Vietnam | East Asia and Pacific | Lower-middle | N/A | N/A | In this paper, the authors provide an overview of the animal and human health systems in Vietnam, as well as past, current and future directions of rabies prevention and control | NTDs |
| Nguyen-Viet (125) | 2022 | Decades of emerging infectious disease, food safety, and antimicrobial resistance response in Vietnam: The role of One Health | Vietnam | East Asia and Pacific | Lower-middle | July 2021 | Workshop | Here, we reflect on the challenges and opportunities of One Health in the context of zoonoses, food safety, and antimicrobial resistance, drawing on a stocktake of One Health training, policy, and research in Vietnam | One Health |
| Nyokabi (126) | 2023 | Implementing a one health approach to strengthen the management of zoonoses in Ethiopia | Ethiopia | Sub-Saharan Africa | Low | Not stated | Semi-structured interviews, participant observation | Taking a qualitative inductive inquiry approach, this study explores the extent of OH implementation in Ethiopia and the impact of such an approach on human, animal and environmental health | One Health |
| OIE (127) | 2019 | Strengthening veterinary services through the OIE PVS pathway | Multiple | Multiple | Multiple | Not Applicable | Not Applicable | The OIE has developed expanded PVS Pathway options to support Member Countries to understand and tailor their engagement based on their own governance and technical priorities | One Health |
| Okello (128) | 2014 | One Health: Past Successes and Future Challenges in Three African Contexts | Multiple (Nigeria, Tanzania, Uganda) | Sub-Saharan Africa | Multiple (Upper-middle income; lower-middle income) | Not stated | Semi-structured interviews | A qualitative case study methodology was used to examine the emerging relationships between international One Health dialogue and its practical implementation in the African health context | One Health |
| Okello (129) | 2015 | Crossing institutional boundaries: Mapping the policy process for improved control of endemic and neglected zoonoses in sub-Saharan Africa | Multiple (Uganda, Nigeria) | Sub-Saharan Africa | Multiple (Lower-middle income; low-income) | Not stated | Semi-structured interviews | To date, analysis of the processes that prioritize, develop and deliver zoonoses control programmes in many low- and middle-income countries is lacking, despite its potential to highlight significant evidence gaps and institutional constraints to the intersectoral approach required for their control. Policy process analysis was conducted with key policy actors within various ministries/institutes in Uganda and Nigeria |  |
| Okia (130) | 2016 | Consolidating tactical planning and implementation frameworks for integrated vector management in Uganda | Uganda | Sub-Saharan Africa | Low | Not stated | Document review, literature review | This paper outlines the processes undertaken to consolidate tactical planning and implementation frameworks for IVM (Integrated vector management) in Uganda | One Health |
| Osman (131) | 2024 | IHR-PVS National Bridging Workshop for Somalia: An interactive and participatory approach for operationalizing the One Health roadmap | Somalia | Sub-Saharan Africa | Low | November 2023 | Workshop | The overall aim of the exercise was to strengthen multisectoral collaboration at the human-animal interface while improving the country's compliance to international standards and regulations | One Health |
| Parodi (132) | 2011 | One health in central Asia. A situational analysis informing the future | Multiple (Kazakhstan, Kyrgyzstan, Tajikistan, Uzbekistan) | Europe and Central Asia | Multiple (Upper-middle income; Lower-middle income) | May - June 2011 | Interviews; OIE PVS; Adaption of Zoonosis and public health sys | Under the One Health initiative in Central Asia, financed by the World Bank, public health and veterinary services gap assessments were undertaken in four countries - Kazakhstan, Kyrgyzstan, Tajikistan and Uzbekistan - to develop a strategy and action plans, which could build capacity to detect, diagnose and prevent or control zoonotic diseases | One Health |
| Qiu (133) | 2024 | Assessment of the Implementation of Pakistan’s National Action Plan on Antimicrobial Resistance in the Agriculture and Food Sectors | Pakistan | South Asia | Lower-middle | October 2022 | Workshop | To improve subsequent iterations, we assessed the implementation of Pakistan's NAP in the agrifood sectors (NAPag) in October 2022, using the Progressive Management Pathway on AMR tool developed by the FAO | AMR |
| Rubin (134) | 2026 | One Health rapid qualitative assessment: Exploring local governance gaps in Tanzania | Tanzania | Sub-Saharan Africa | Lower-middle | 2023 - 2025 | Focus group and semi-structured interviews | This paper presents findings from an OH-RQA in Tanzania. | One Health |
| Rubin (135) | 2014 | Making one health a reality - Crossing Bureaucratic boundaries | Multiple | Multiple | Multiple | Not Stated | Not Stated | This article reviews interagency One Health collaborations, nationally and internationally. It presents a series of case studies that describe situations in which barriers were overcome, thus culminating in successful One Health outcomes. | One Health |
| Sande (136) | 2019 | Getting ready for integrated vector management for improved disease prevention in Zimbabwe: A focus on key policy issues to consider | Zimbabwe | Sub-Saharan Africa | Lower-middle | N/A | N/A | This paper outlines Zimbabwe's potential readiness in harnessing integrated vector management (IVM) strategy for enhanced control of vector-borne diseases. The objective is to provide guidance for the country in the implementation of the national IVM strategy in order to make improvements required in thematic areas of need | Other |
| Schneider (137) | 2014 | OIE PVS Evaluation report of the veterinary services of Brazil | Brazil | Latin America and the Caribbean | Upper-middle | February 2014 | Meetings and OIE PVS evaluation | The objective and scope of the OIE PVS Evaluation includes all aspects relevant to the OIE Terrestrial Animal Health Code and the quality of Veterinary Services | One Health |
| Shabangu (138) | 2025 | Policy makers’ perceptions on implementation of National Action Plans on antimicrobial resistance in South Africa and Eswatini using coordination, accountability, resourcing, regulation and ownership framework (2018-2019) | Multiple (South Africa, Eswatini) | Sub-Saharan Africa | Multiple (Upper-middle; lower-middle) | November 2018 – March 2019 | Semi-structured interviews | This study provides insights into the perspectives of policymakers in South Africa and Eswatini regarding the effectiveness, gaps, and challenges associated with NAPs on AMR implementation, using the CARRO framework as a guiding tool. | AMR |
| Shabangu (139) | 2023 | Barriers to implementing National Action Plans on antimicrobial resistance using a One Health Approach: policymakers' perspectives from South Africa and Eswatini | Multiple (South Africa, Eswatini) | Sub-Saharan Africa | Multiple (Upper-middle; lower-middle) | November 2018 - March 2019 | Semi-structured interviews | This study aimed to explore policymakers experiences on barriers to implementing National Action Plans on antimicrobial resistance using a One Health approach in South Africa and Eswatini | AMR |
| Shiferaw (140) | 2017 | Frameworks for preventing, detecting, and controlling zoonotic diseases | Multiple (Ethiopia, Democratic Republic of the Congo, Georgia) | Multiple (Sub-Saharan Africa, Europe and Central Asia) | Multiple (upper-middle income; low-income) | Not Stated | Not Stated | We highlight 3 examples of approaches to implement zoonotic disease prevention and control programs. Rabies control in Ethiopia using an umbrella approach: A monkeypox program in Democratic Republic of the Congo implemented using a stepwise manner: The third, a pathogen discovery program applied in the country of Georgia | One Health |
| Smith (141) | 2015 | One World-One Health and neglected zoonotic disease: Elimination, emergence, and emergency in Uganda | Uganda | Sub-Saharan Africa | Low | February - April 2013 | Semi-structured interviews | The article presents three dimensions of intervention and interaction, each of which connects to an OWOH narrative. Firstly, the paper considers the global politics of health prioritisation and how an emergent NTD lobby has grown in response. Secondly, we present the Stamp Out Sleeping Sickness (SOS) campaign. Thirdly, we describe an external 'emergency response' that took place towards Uganda's border as infections rose | NTDs |
| Sow (142) | 2026 | Governing antibiotic resistance through One Health: Insights from the political and legal landscape in Senegal | Senegal | Sub-Saharan Africa | Lower-middle | January 2023 – August 2023 | Document review and semi-structured interviews | This study aims to examine the capacity of the Senegalese politico-institutional framework to manage the ABR issue from a One Health perspective. | AMR |
| Sow (143) | 2025 | Supporting One Health policies to manage antibiotic resistance in Senegal: A systems analysis using group model building | Senegal | Sub-Saharan Africa | Lower-middle | October 2023 | Group model building workshop | The aims of this study were to: (i) map the ABR dynamics in Senegal and identify important feedback loops; and (ii) identify priority policy actions to mitigate ABR in the country | AMR |
| Standley (144) | 2019 | Assessing health systems in Guinea for prevention and control of priority zoonotic diseases: A One Health approach | Guinea | Sub-Saharan Africa | Lower-middle | September 2015 - April 2016 | Multiple (Workshops, document review, site visits, interviews) | To guide One Health capacity building efforts in the Republic of Guinea in the wake of the 2014–2016 Ebola virus disease (EVD) outbreak, we sought to identify and assess the existing systems and structures for zoonotic disease detection and control | One Health |
| Stewart-Ibarra (145) | 2019 | Co-developing climate services for public health: stakeholder needs and perceptions for the prevention and control of Aedes-transmitted diseases in the Caribbean | Caribbean | Multiple | Multiple | April - June 2017 | Interviews, surveys, and national workshops with stakeholders | The objective of this study was to identify health and climate stakeholder perceptions and needs in the Caribbean, with respect to the development of climate services for arboviruses | NTDs |
| Sumpradit (146) | 2021 | Thailand's national strategic plan on antimicrobial resistance: progress and challenges | Thailand | East Asia and Pacific | Upper-middle | N/A | N/A | We outline here the progress and challenges of implementing Thailand's national strategic plan on antimicrobial resistance from 2017 until the middle of 2021. We also discuss interim outcomes and share lessons learnt | AMR |
| Tangcharoensathien (147) | 2017 | Antimicrobial resistance: from global agenda to national strategic plan, Thailand | Thailand | East Asia and Pacific | Upper-middle | N/A | N/A | On the basis of local evidence and with the strong participation of relevant stakeholders, the first national strategic plan on antimicrobial resistance has been developed in Thailand | AMR |
| Tegegne (148) | 2024 | Implementation of one health surveillance systems: Opportunities and challenges - lessons learned from the OH-EpiCap application | Multiple | Europe and Central Asia | High | April - November 2022 | National multi-sectoral surveillance systems | In this study, we assessed the OH practices of foodborne and other zoonotic hazards surveillance systems in several European countries to identify the main barriers that contribute to sub-optimal OH functioning | One Health |
| Tiensin (149) | 2015 | How can we progress the cooperation between animal health sector and public health sector | Thailand | East Asia and Pacific | Upper-Middle | 2012 - 2014 | IHR assessment, OIE PVS, PVS Gap analysis | This paper aims to increase awareness and understanding of the WHO IHR Monitoring Framework (IHRMF) and the OIE PVS Pathway to review and evaluate frameworks and tools of the IHRMF and the OIE PVS pathway to help assess the capacities of the human and animal health sectors | One Health |
| Time (150) | 2024 | From ideal to reality: governance of AMR in a multi-level setting | Norway | Europe and Central Asia | High | 2017-2024 | Document review, semi-structured interviews | This paper asks whether, and if so how, it is possible to design a system characterised by coordination across sectors and levels of governance aimed at governing AMR with a focus on Norway. | AMR |
| Travis (151) | 2014 | One Health: Lessons Learned from East Africa | East Africa | Multiple | Multiple | Not stated | N/A | The following are a few initiatives and/or lessons learned from East Africa as experienced by the authors | One Health |
| Valenzuela (152) | 2025 | Situational analysis of antimicrobial resistance policies and program implementation in the Philippines, 2019-2023 | Philippines | East Asia and Pacific | Lower-middle | Not stated | Self-assessment worksheets, document review, and interviews | This study serves as a situational analysis of the current AMR policy and programming in the Philippines, assessing the implementation status of the second iteration of the PNAP to Combat AMR (2019-2023) | AMR |
| Wakimoto (153) | 2022 | COVID-19 and zoonoses in Brazil: Environmental scan of one health preparedness and response | Brazil | Latin America and the Caribbean | Upper-middle | October 2020 | Environmental scan | We aimed to draw on Brazilian preparedness and response to COVID-19 and zoonoses to assess if OH principles and equity considerations influence health policy response during infectious disease outbreaks | One Health |
| Wallinga (154) | 2022 | A Review of the Effectiveness of Current US Policies on Antimicrobial Use in Meat and Poultry Production | USA | North America | High | Not stated | Not stated | Here, we describe legislative and regulatory efforts at different levels of governance in the USA, to curtail food animal consumption of medically important antimicrobials | AMU |
| Weaver (155) | 2019 | OIE PVS Evaluation Follow-up Mission Report: Rwanda | Rwanda | Sub-Saharan Africa | Low | July 2019 | Meetings and OIE PVS evaluation | The objective and scope of the OIE PVS Evaluation covers all aspects of the veterinary domain relevant to the OIE Terrestrial Animal Health Code and the quality of Veterinary Services | One Health |
| Wignjadiputro (156) | 2020 | Whole–of–society approach for influenza pandemic epicenter Containment exercise in Indonesia | Indonesia | East Asia and Pacific | Upper-middle | 2017 | Simulation exercise | This article aims to describe lesson learned from the full scale influenza pandemic simulation, conducted by the MoH of the Republic of Indonesia, in collaboration with the WHO in 2017. | Avian Influenza |
| Woolaston (157) | 2022 | An argument for pandemic risk management using a multidisciplinary One Health approach to governance: an Australian case study | Australia | East Asia and Pacific | High | July - August 2020 | Workshop | We discuss the adoption of a comprehensive and interdisciplinary 'One Health' approach to pandemic risk management in Australia. | One Health |
| World Bank (158) | 2022 | Reducing pandemic risks at source: Wildlife, environment and One Health foundations in East and South Asia | Multiple | Multiple (East Asia and Pacific, South Asia) | Multiple (high-income; Low-income) | Not Applicable | Not Applicable | This report includes detailed findings and recommendations across the entire span of activities to monitor and respond to EIDs of wildlife origin | One Health |
| World Bank; World Health Organization (159) | 2022 | Sustaining Action Against Antimicrobial Resistance: A case series of country experiences | Multiple (Sierra Leone, Burkina Faso, Malawi, Jordan) | Multiple (Middle East and North Africa, Sub-Saharan Africa) | Multiple (Lower middle income; low income) | Not stated | Not stated | The following case series showcase recent country experiences while developing and implementing their NAPs for AMR | AMR |
| World Health Organization (160) | 2018 | Tackling antimicrobial resistance (AMR) Together: Working Paper 1.0: Multisectoral coordination | Multiple | Multiple | Multiple | Not Applicable | Not Applicable | This working paper was conceived to offer practical tips and suggestions on how to establish and sustain the multisectoral collaboration needed to develop and implement National Action Plans on AMR (NAPs). | AMR |
| World Health Organization (161) | 2022 | WHO implementation handbook for national action plans on antimicrobial resistance: Guidance for the human health sector | Multiple | Multiple | Multiple | Not Applicable | Not Applicable | The handbook is primarily intended to guide implementation of NAPs on AMR in the human health sector following a six-step continuous process | AMR |
| World Health Organization (162) | 2018 | Resource mobilisation for AMR: Getting AMR into plans and budgets of government and development partners | Nepal | South Asia | Lower-middle | December 2017 | Interviews, focus groups, document review | The overall objectives of the work conducted in Nepal, on enhancing investment in AMR, are: (1) to assist teams working on AMR in Nepal to explore the scope to scale up delivery of AMR activities through integration within existing programmes and projects, including those under development. (2) To identify any funders with an interest in funding AMR-related work. (3) To understand how development partners view efforts to mobilize resources for AMR in the country | AMR |
| World Health Organization (163) | 2014 | WHO-OIE Operational Framework for good governance at the human-animal interface: Bridging WHO and OIE tools for the assessment of national capacities | Multiple | Multiple | Multiple | Not Applicable | Not Applicable | The objective of this Operational Framework is to help Member Countries contribute to the development of a coherent system of global health governance at the human-animal interface | One Health |
| World Health Organization (164) | 2018 | Viet Nam Mission Report | Vietnam | East Asia and Pacific | Lower-middle | May 2018 | Interviews, site visits, document review, roundtable discussion | Specific aims were to study WHO's role in the country's approach to health security, to better understand the key elements of Viet Nam's impressive progress, and to extract lessons from its experience with the Joint external Evaluations (JEEs) and National Action Plan | One Health |
| Yambayamba (165) | 2024 | Learning from over ten years of implementing the One Health approach in the Democratic Republic of Congo: A qualitative study | Democratic Republic of Congo | Sub-Saharan Africa | Low | June 2023 - Jan 2024 | Document review; semi-structured interviews | This study investigates OH institutionalization and implementation in the DRC, describes the process of OH decentralization, and identifies the opportunities and challenges of sustaining these efforts | One Health |
| Yang (166) | 2024 | Antimicrobial resistance in China across human, animal, and environment sectors - A review of policy documents using a governance framework | China | East Asia and Pacific | Upper-middle | January 2023 | Document review, semi-structured interviews, survey data | This study aims to review the content of AMR policy documents at the national level using a governance framework covering three areas: Policy Design; Implementation tools; and Monitoring and Evaluation | AMR |
| Yasobant (167) | 2020 | ‘One Health’ Actors in Multifaceted Health Systems: An Operational Case for India | India | South Asia | Lower-middle | September 2018 - October 2019 | Semi-structured interviews, network analysis | The overall aim of this study is to identify and categorize actors at the human-animal health system interface and attempted to document the issues and challenges pertaining to the ISC in two different situations (one during an outbreak and another during non-outbreak) with a focus on prevention and control of zoonotic diseases in Ahmedebad, India | One Health |
| Yasobant (168) | 2021 | Systemic factors for enhancing intersectoral collaboration for the operationalization of One Health: A case study in India | India | South Asia | Lower-middle | July - October 2019 | Vignette interview, Delphi process, participatory workshop | This study documented and validated the innovative strategy for intersectoral collaboration, focusing on effectual prevention and control of zoonotic diseases with its enabling factors for a city in western India, Ahmedabad | One Health |
| Yopa (169) | 2023 | Barriers and enablers to the implementation of the one health strategies in developing countries: A systematic review | Multiple | Multiple | Multiple (Upper-middle; Lower-middle-; Low) | 2008 - 2023 | Systematic review | This systematic review aimed to identify barriers and enablers in implementing OH strategies in LMICs. The findings of this review will help identify the root of the matter globally and inform us on the potential actions to initiate | One Health |
| Zaidi (170) | 2015 | A Collaborative Initiative For the Containment of Antimicrobial Resistance in Mexico | Mexico | Latin America and the Caribbean | Upper-middle | N/A | N/A | This study describes the process and outcomes involved in the development of this endeavor. We consider the challenges encountered by this initiative as well as its potentialities and draw lessons for Mexico and other countries seeking to establish national strategies for improved antimicrobial use and containment of resistance | AMU |
| Zhang (171) | 2025 | Integrating One Health governance in China: Assessing structural implementation and operational entry points | China | East Asia and Pacific | Upper-middle | January 2024 – April 2024 | Literature review and expert interviews | This study evaluates China’s One Health governance through four structural domains – Monitoring & Evaluation, Intervention & Response, Surveillance & Early Warning, and Capacity Building – and four operational entry points – Technology, Information, Human Resources, and Finance. The aim is to identify strengths, gaps, and provide actionable recommendations for improvement | One Health |

**References:**

1. Abass K, Hinkston I, Yusuf H, Semerjian L. One health in Gulf Cooperation Council countries: A roadmap for integrated human–animal–environment health. J Public Health. 2025 Nov 28. doi:10.1007/s10389-025-02621-y

2. Abutarbush SM, Hamdallah A, Hawawsheh M, Alsawalha L, Elizz NA, Dodeen R. Implementation of One Health approach in Jordan: Review and mapping of ministerial mechanisms of zoonotic disease reporting and control, and inter-sectoral collaboration. One Health. 2022;15:100406. doi:10.1016/j.onehlt.2022.100406

3. Abuzerr S, Zinszer K, Assan A. Implementation challenges of an integrated One Health surveillance system in humanitarian settings: A qualitative study in Palestine. SAGE Open Med. 2021;9. doi:10.1177/20503121211043038

4. Acharya KP, Karki S, Shrestha K, Kaphle K. One health approach in Nepal: Scope, opportunities and challenges. One Health. 2019 Dec;8:100101. doi:10.1016/j.onehlt.2019.100101

5. Adewumi IP, Adeyemi QO, Babatope AE, Ajisafe DO, Adepoju KO. Intersectoral collaboration for strengthening infectious disease prevention and control in Nigeria: a narrative review. Discov Public Health. 2026 Jan 6;23(1):19. doi:10.1186/s12982-025-01220-2

6. Adini B, Singer SR, Ringel R, Dickmann P. Earlier detection of public health risks – Health policy lessons for better compliance with the International Health Regulations (IHR 2005): Insights from low-, mid- and high-income countries. Health Policy. 2019;123(10):941–6. doi:10.1016/j.healthpol.2019.06.007

7. Adnyana IM, Utomo B, Eljatin DS, Sudaryati NL. One Health approach and zoonotic diseases in Indonesia: Urgency of implementation and challenges. Narra J. 2023;3(3):e257. doi:10.52225/narra.v3i3.257

8. Africa Centres for Disease Control and Prevention (CDC). Africa CDC AMR [Internet]. Available from: https://africacdc.org/wp-content/uploads/2024/08/African-Union-AMR-Landmark-Report-.pdf

9. Aggarwal D, Ramachandran A. One Health Approach to Address Zoonotic Diseases. Indian J Community Med. 2020;45(Suppl 1):S6–8. doi:10.4103/ijcm.IJCM_398_19

10. Aguiar R, Ruckert A, Harris F, Boudreau LeBlanc A, Carmo LP, Davies-Venn M, et al. Equity in the governance of antimicrobial resistance surveillance: Global experts’ perspectives. Soc Sci Med. 2025 Dec;387:118702. doi:10.1016/j.socscimed.2025.118702

11. Aguiar R, Keil R, Gray R, Wiktorowicz M. One health governance of antimicrobial resistance seen through an Urban Political Ecology lens: a critical interpretive synthesis. Crit Public Health. 2024 Dec 31;34(1):1–23. doi:10.1080/09581596.2024.2395825

12. Ahmed SM, Naher N, Tune SNBK, Islam BZ. The Implementation of National Action Plan (NAP) on Antimicrobial Resistance (AMR) in Bangladesh: Challenges and Lessons Learned from a Cross-Sectional Qualitative Study. Antibiotics. 2022;11(5). doi:10.3390/antibiotics11050690

13. Allal L, Mahrous H, Saad A, Refaei S, Attia M, Mahrous I, et al. From Four-Way Linking to a One Health Platform in Egypt: institutionalisation of a multidisciplinary and multisectoral One Health system. Rev Sci Tech Int Off Epizoot. 2019;38(1):261–70. doi:10.20506/rst.38.1.2958

14. Allel K, Fernandez-Miyakawa M, Gaze W, Petroni A, Corso A, Luna F, et al. Opportunities and challenges in antimicrobial resistance policy including animal production systems and humans across stakeholders in Argentina: a context and qualitative analysis. Sota RL GS Bruni SS, Reyher K, West H, Davies P, Moran D, AMR Policy Research Group Argentina, editors. BMJ Open. 2024;14(6):e082156. Located at: Ovid MEDLINE(R). doi:10.1136/bmjopen-2023-082156

15. Allen HA. Governance and one health: Exploring the impact of federalism and bureaucracy on zoonotic disease detection and reporting. Vet Sci. 2015;2(2):69–83. doi:10.3390/vetsci2020069

16. Anderson M, Clift C, Schulze K, Sagan A, Nahrgang S, Ait Ouakrim D, et al. Averting the AMR crisis: What are the avenues for policy action for countries in Europe? Eur Obs Health Syst Policies. 2019.

17. Asaaga FA, Young JC, Oommen MA, Chandarana R, August J, Joshi J, et al. Operationalising the “One Health” approach in India: facilitators of and barriers to effective cross-sector convergence for zoonoses prevention and control. BMC Public Health. 2021 Dec;21(1):1517. doi:10.1186/s12889-021-11545-7

18. Ayobami O, Mark G, Kadri-Alabi Z, Achi CR, Jacob JC. COVID-19: an opportunity to re-evaluate the implementation of a One Health approach to tackling emerging infections in Nigeria and other sub-Saharan African countries. J Egypt Public Health Assoc. 2021;96(1). doi:10.1186/s42506-021-00085-y

19. Bakiika H, Obuku EA, Bukirwa J, Nakiire L, Robert A, Nabatanzi M, et al. Contribution of the one health approach to strengthening health security in Uganda: a case study. BMC Public Health. 2023;23(1):1498. doi:10.1186/s12889-023-15670-3

20. Barroga TR, Gordoncillo MJ, Lagayan MG, Bernales R, Caniban M, Lopez E, et al. Practical inter-sectoral linking: Tool to rabies One Health coordination to the grass-roots level. Zoonoses Public Health. 2018;65(7):805–14. doi:10.1111/zph.12502

21. Batsukh Z, Tsolmon B, Otgonbaatar D, Undraa B, Dolgorkhand A, Ariuntuya O. One health in Mongolia. Curr Top Microbiol Immunol. 2013;366:123–37. doi:10.1007/82_2012_253

22. Belot G, Caya F, Errecaborde KM, Traore T, Lafia B, Skrypnyk A, et al. IHR-PVS National Bridging Workshops, a tool to operationalize the collaboration between human and animal health while advancing sector-specific goals in countries. PLOS ONE. 2021 Jun 1;16(6):e0245312. doi:10.1371/journal.pone.0245312

23. Beltramo B, Kolluru S, Slager L, Wall L, Ostwald K, Rasali D. Comparative Analysis of One Health Policies in Asia for Exploring Opportunities for British Columbia in Canada. Int J Environ Res Public Health. 2024 Dec 30;22(1):34. doi:10.3390/ijerph22010034

24. Berman TS, Barnett-Itzhaki Z, Berman T, Marom E. Antimicrobial resistance in food-producing animals: towards implementing a one health based national action plan in Israel. Isr J Health Policy Res. 2023;12(1). doi:10.1186/s13584-023-00562-z

25. Beyene AM, Andualem T, Dagnaw GG, Getahun M, LeJeune J, Ferreira JP. Situational analysis of antimicrobial resistance, laboratory capacities, surveillance systems and containment activities in Ethiopia: A new and one health approach. One Health. 2023;16. doi:10.1016/j.onehlt.2023.100527

26. Bhat P, S JH, Raju MK, Sooda S, K P, Kumar R. Kyasanur Forest Disease, is our surveillance system healthy to prevent a larger outbreak? A mixed-method study, Shivamogga, Karnataka, India: 2019. Int J Infect Dis. 2021;110 Suppl 1:S50–61. doi:10.1016/j.ijid.2021.07.076

27. Björkman I, Röing M, Sternberg Lewerin S, Stålsby Lundborg C, Eriksen J. Animal Production With Restrictive Use of Antibiotics to Contain Antimicrobial Resistance in Sweden—A Qualitative Study. Front Vet Sci. 2021 Jan 15;7:619030. doi:10.3389/fvets.2020.619030

28. Björkman I, Röing M, Eriksen J, Stålsby Lundborg C. Swedish Efforts to Contain Antibiotic Resistance in the Environment-A Qualitative Study among Selected Stakeholders. Antibiot Basel. 2022;11(5). doi:10.3390/antibiotics11050646

29. Blankart CR, De Gani SM, Crimlisk H, Desmedt M, Bauer B, Doyle G. Health literacy, governance and systems leadership contribute to the implementation of the One Health approach: a virtuous circle. Health Policy. 2024;143:105042. doi:10.1016/j.healthpol.2024.105042

30. Bond KC, Macfarlane SB, Burke C, Ungchusak K, Wibulpolprasert S. The evolution and expansion of regional disease surveillance networks and their role in mitigating the threat of infectious disease outbreaks. Emerg Health Threats J. 2013;6. doi:10.3402/ehtj.v6i0.19913

31. Bordier M, Binot A, Pauchard Q, Nguyen DT, Trung TN, Fortané N, et al. Antibiotic resistance in Vietnam: moving towards a One Health surveillance system. BMC Public Health. 2018 Sep 24;18(1):1136. doi:10.1186/s12889-018-6022-4

32. Bronzwaer S, de Coen W, Heuer O, Marnane I, Vidal A. The framework for action of the Cross-agency One Health Task Force. One Health. 2024;19:100925. doi:10.1016/j.onehlt.2024.100925

33. Brunskill I, Somanader DS, Perrin M, Barkema HW, Hillier S, Hindmarch S, et al. Seeing the whole elephant: designing “one health” governance to fight antimicrobial resistance. Clin Microbiol Infect Off Publ Eur Soc Clin Microbiol Infect Dis. 2024 Apr;30(4):419–22. doi:10.1016/j.cmi.2023.09.021 PubMed PMID: 37802304.

34. Buregyeya E, Atusingwize E, Nsamba P, Musoke D, Naigaga I, Kabasa JD, et al. Operationalizing the One Health Approach in Uganda: Challenges and Opportunities. J Epidemiol Glob Health. 2020;10(4):250–7. doi:10.2991/jegh.k.200825.001

35. Busani L, Creti R, Fabbro E, Prestinaci F, Pantosti A, Marella AM, et al. Fighting Antimicrobial Resistance and Healthcare-Associated Infections in EU-JAMRAI: The One-Health Response from Italy. Chemotherapy. 2024;69(1):56–64. doi:10.1159/000531684

36. Cediel Becerra NM, Olaya Medellin AM, Tomassone L, Chiesa F, De Meneghi D. A Survey on One Health Approach in Colombia and Some Latin American Countries: From a Fragmented Health Organization to an Integrated Health Response to Global Challenges. Front Public Health. 2021;9. doi:10.3389/fpubh.2021.649240

37. Cham D, Barrow A, Shah-Rohlfs R, Standley CJ. Can global health security frameworks measure One Health implementation in West Africa?A mixed-methods study. BMC Public Health. 2024;24(1):2113. doi:10.1186/s12889-024-19617-0

38. Chanda E, Masaninga F, Coleman M, Sikaala C, Katebe C, MacDonald M, et al. Integrated vector management: The Zambian experience. Malar J. 2008;7. doi:10.1186/1475-2875-7-164

39. Chatterjee P, Kakkar M, Chaturvedi S. Integrating one health in national health policies of developing countries: India’s lost opportunities. Infect Dis Poverty. 2016;5(1). doi:10.1186/s40249-016-0181-2

40. Chattopadhyay K, Fournié G, Abul Kalam Md, Biswas PK, Hoque A, Debnath NC, et al. A Qualitative Stakeholder Analysis of Avian Influenza Policy in Bangladesh. EcoHealth. 2018 Mar;15(1):63–71. doi:10.1007/s10393-017-1285-2

41. Chen W, Zeng Y, Zheng J, Wang J, Gu W, Li M, et al. Evaluation of antimicrobial resistance governance across 193 countries to inform the 2026 Global Action Plan update. Nat Med. 2026 Mar 3. doi:10.1038/s41591-026-04257-1

42. Cheptoyek L, Kikuvi G, Gachohi J. Utilization and associated determinants of multi-sectoral approach in zoonotic disease surveillance among animal and human healthcare workers in Nakuru County, Kenya. Open Res Eur. 2024;4:103. Located at: Dryad/10.5061/dryad.g1jwstqzm. doi:10.12688/openreseurope.17583.2

43. Chongo I, Tivane A, Monteiro V, Inlamea O, Maholela P, Nhanombe I, et al. Outcomes from a Zoonotic Disease Prioritization workshop using One Health approach in Mozambique, 2018 to 2023. ONE Health OUTLOOK. 2024;6(1). Located at: WOS:001322649200001. doi:10.1186/s42522-024-00113-9

44. Chua AQ, Verma M, Hsu LY, Legido-Quigley H. An analysis of national action plans on antimicrobial resistance in Southeast Asia using a governance framework approach. Lancet Reg Health West Pac. 2021 Feb;7:100084. doi:10.1016/j.lanwpc.2020.100084 PubMed PMID: 34327414; PubMed Central PMCID: PMC8315476.

45. Chua AQ, Verma M, Azupardo K, Lota MM, Hsu LY, Legido-Quigley H. A Qualitative Study on the Policy Process and Development of the National Action Plan on Antimicrobial Resistance in Singapore. Antibiotics. 2023 Aug 16;12(8):1322. doi:10.3390/antibiotics12081322 PubMed PMID: 37627742; PubMed Central PMCID: PMC10451339.

46. Chunsuttiwat S. Response to avian influenza and preparedness for pandemic influenza: Thailand’s experience. Respirology. 2008;13 Suppl 1:S36-40. doi:10.1111/j.1440-1843.2008.01256.x

47. Collineau L, Rousset L, Colomb-Cotinat M, Bordier M, Bourely C. Moving towards One Health surveillance of antibiotic resistance in France: a semi-quantitative evaluation of the level of collaboration within the national surveillance system. JAC-Antimicrob Resist. 2024 Feb 1;6(1):dlae008. doi:10.1093/jacamr/dlae008

48. Corrêa JS, Zago LF, Da Silva-Brandão RR, de Oliveira SM, Fracolli LA, Padoveze MC, et al. The governance of antimicrobial resistance in Brazil: Challenges for developing and implementing a one health agenda. Glob Public Health. 2023 Jan;18(1):2190381. doi:10.1080/17441692.2023.2190381 PubMed PMID: 36934430.

49. Davido B, Ny S, Van Lingen C, Årdal C, Alonso Irujo L, Linnros S, et al. Strengthening antimicrobial resistance governance in Europe: a coordinated one health approach. Lancet Reg Health - Eur. 2026 Feb;61:101540. doi:10.1016/j.lanepe.2025.101540

50. Dayapera LZA, Sy JCY, Valenzuela S, Eala SJL, Del Rosario CMIP, Buensuceso KNC, et al. One health in the Philippines: A review and situational analysis. One Health Amst Neth. 2024;18(101660501):100758. Located at: Ovid MEDLINE(R) PubMed-not-MEDLINE. doi:10.1016/j.onehlt.2024.100758

51. de Best PA, Broekhuizen H, Sikkema RS, Koopmans MPG, Timen A. One Health preparedness and response for mosquito-borne viruses: a stakeholder- and social network-analysis in the Netherlands. BMC Public Health. 2025;25(1):307. doi:10.1186/s12889-025-21539-4

52. de la Rocque S, Errecaborde KMM, Belot G, Brand T, Shadomy S, von Dobschuetz S, et al. One health systems strengthening in countries: Tripartite tools and approaches at the human-animal-environment interface. BMJ Glob Health. 2023;8(1). doi:10.1136/bmjgh-2022-011236

53. Degeling C, Johnson J, Kerridge I, Wilson A, Ward M, Stewart C, et al. Implementing a One Health approach to emerging infectious disease: reflections on the socio-political, ethical and legal dimensions. BMC Public Health. 2015;15:1307. doi:10.1186/s12889-015-2617-1

54. Delpy L, Astbury CC, Aenishaenslin C, Ruckert A, Penney TL, Wiktorowicz M, et al. Integrated surveillance systems for antibiotic resistance in a One Health context: a scoping review. BMC Public Health. 2024 Jun 27;24(1):1717. doi:10.1186/s12889-024-19158-6

55. Denis-Robichaud J, Hindmarch S, Nswal NN, Mutabazi JC, D’Astous M, Gangbè M, et al. One Health communication channels: a qualitative case study of swine influenza in Canada in 2020. BMC Public Health. 2024;24(1):964. doi:10.1186/s12889-024-18460-7

56. Deruelle T. A tribute to the foot soldiers: European health agencies in the fight against antimicrobial resistance. Health Econ Policy Law. 2021;16(1):23–37. doi:10.1017/S1744133120000213

57. Don Bamunusinghage Nihal P, Dangolla A, Hettiarachchi R, Abeynayake P, Stephen C. CHALLENGES AND OPPORTUNITIES FOR WILDLIFE DISEASE SURVEILLANCE IN SRI LANKA. J Wildl Dis. 2020;56(3):538–46. doi:10.7589/2019-07-181

58. El-Jardali F, Fadlallah R, Daher N. Multi-sectoral collaborations in selected countries of the Eastern Mediterranean region: assessment, enablers and missed opportunities from the COVID-19 pandemic response. Health Res Policy Syst. 2024;22(1). doi:10.1186/s12961-023-01098-z

59. Erkyihun GA, Gari FR, Edao BM, Kassa GM. A review on One Health approach in Ethiopia. One Health Outlook. 2022;4(1):8. doi:10.1186/s42522-022-00064-z

60. Espeschit IF, Santana CM, Moreira MAS. Public Policies and One Health in Brazil: The Challenge of the Disarticulation. Front Public Health. 2021;9:644748. doi:10.3389/fpubh.2021.644748

61. European Commission. Directorate General for Health and Food Safety. Member States’ One Health National Action Plans against antimicrobial resistance: overview report. [Internet]. LU: Publications Office; 2022 [cited 2025 Jul 11]. Available from: https://data.europa.eu/doi/10.2875/152822

62. FAO, OIE, World Health Organization, UN System Influenza Coordinator (UNSIC), UNICEF, World Bank. Contributing to One World, One Health: A strategic Framework for Reducing Risks of Infectious DIseases at the Animal-Human-Ecosystems Interface. 2008.

63. Food and Agriculture Organization (FAO). One Health: Seeing around corners. A regional communication strategy framework against infectious diseases in Asia and the Pacific 2011-2016. 2011 Aug;72.

64. Food and Agriculture Organization (FAO), editor. Fourth report on the Global Programme for the Prevention and Control of HPAI (January - December 2010). Rome: FAO; 2011.

65. FAO, editor. Fifth Report on the Global Programme for the Prevention and Control of HPAI (January 2011 - January 2012). Rome: FAO; 2013. 127 p.

66. Food and Agriculture Organization of the United Nations, International Office of Epizootics, World Health Organization, editors. Influenza and other emerging zoonotic diseases at the human-animal interface. Rome, Italy: Published by the Food and Agriculture Organization of the United Nations and the World Organisation for Animal Health and the World Health Organization; 2011. 53 p. (FAO animal production and health proceedings; no. 13).

67. World Health Organization, Food and Agriculture Organization of the United Nations, United Nations Environment Programme, World Organisation for Animal Health. A guide to implementing the One Health Joint Plan of Action at national level [Internet]. Geneva; 2023 [cited 2024 Dec 13]. Available from: https://iris.who.int/bitstream/handle/10665/374825/9789240082069-eng.pdf?sequence=1

68. World Health Organization, Food and Agriculture Organization of the United Nations, World Organisation for Animal Health. Taking a multisectoral, one health approach: a tripartite guide to addressing zoonotic diseases in countries [Internet]. Geneva: World Health Organization; 2019 [cited 2025 Jul 14]. 151 p. Available from: https://iris.who.int/handle/10665/325620

69. Farag EAB, Nour M, El Idrissi A, Berrada J, Moustafa A, Mehmood M, et al. Survey on Implementation of One Health Approach for MERS-CoV Preparedness and Control in Gulf Cooperation Council and Middle East Countries. Emerg Infect Dis. 2019;25(3). doi:10.3201/eid2503.171702

70. Feng X, Jiang N, Zheng J, Zhu Z, Chen J, Duan L, et al. Advancing knowledge of One Health in China: lessons for One Health from China’s dengue control and prevention programs. Sci One Health. 2024;3:100087. doi:10.1016/j.soh.2024.100087

71. Fotakis EA, Papamichail D, Boutsini S, Patsoula E, Panagiotopoulos T. Leishmaniasis in Greece: Prospects of transitioning to a One Health surveillance system. One Health. 2024;19. doi:10.1016/j.onehlt.2024.100896

72. Frumence G, Mboera LEG, Sindato C, Katale BZ, Kimera S, Metta E, et al. The governance and implementation of the national action plan on antimicrobial resistance in Tanzania: A qualitative study. Antibiotics. 2021;10(3). doi:10.3390/antibiotics10030273

73. Frumence G, Mboera LEG, Katale BZ, Sindato C, Kimera S, Durrance-Bagale A, et al. Policy actors and human and animal health practitioners’ perceptions of antimicrobial use and resistance in Tanzania: A qualitative study. J Glob Antimicrob Resist. 2021 Jun;25:40–7. doi:10.1016/j.jgar.2021.02.027

74. Gao Y, Li C, Zhou Q, Hao M. The application of One Health concept in China and its practice and innovation in cross-sector cooperation. Public Health Pract. 2025 Jun;9:100574. doi:10.1016/j.puhip.2024.100574

75. Gautier A, Gardon S, Déprés C. The emergence of the Biodiversity/Health nexus: making biodiversity a health issue. Rev Agric Food Env Stud. 2023;104(1):27–46. doi:10.1007/s41130-023-00189-3

76. Gebrekidan TK, Weldemariam NG, Hidru HD, Gebremedhin GG, Weldemariam AK. Impact of improper municipal solid waste management on fostering One Health approach in Ethiopia - challenges and opportunities: A systematic review. Sci One Health. 2024;3:100081. doi:10.1016/j.soh.2024.100081

77. Glover RE, Naylor NR. The WHO costing and budgeting tool for national action plans on antimicrobial resistance-a practical addition to the WHOle toolkit. JAC Antimicrob Resist. 2023;5(3):dlad064. doi:10.1093/jacamr/dlad064

78. Gobena A, Bullon C, Viinikainen T. Regulatory frameworks to address antimicrobial resistance in the food and agriculture sectors [Internet]. FAO Legislative Studies; 2024 [cited 2025 Jul 11]. Available from: https://openknowledge.fao.org/handle/20.500.14283/cd1272en doi:10.4060/cd1272en

79. Goryoka G, Lokossou V, Varela K, Oussayef N, Kofi B, Iwar V, et al. Prioritizing zoonotic diseases using a multisectoral, One Health approach for The Economic Community of West African States (ECOWAS). ONE Health OUTLOOK. 2021;3(1). Located at: WOS:000721956400001. doi:10.1186/s42522-021-00055-6

80. Grant C, Anderson N, Machila N. Stakeholder Narratives on Trypanosomiasis, Their Effect on Policy and the Scope for One Health. PLoS Negl Trop Dis. 2015;9(12):e0004241. doi:10.1371/journal.pntd.0004241

81. Griffiths K, Banjara MR, O’Dempsey T, Munslow B, Kroeger A. Public health responses to a dengue outbreak in a fragile state: a case study of Nepal. J Trop Med. 2013;2013:158462. doi:10.1155/2013/158462

82. Halabi SF. Adaptation of Animal and Human Health Surveillance Systems for Vector-Borne Diseases Accompanying Climate Change. J Law Med Ethics. 2020;48(4):694–704. doi:10.1177/1073110520979375

83. Häsler B, Bazeyo W, Byrne AW, Hernandez-Jover M, More SJ, Rüegg SR, et al. Reflecting on One Health in Action During the COVID-19 Response. Front Vet Sci. 2020;7:578649. doi:10.3389/fvets.2020.578649

84. Hassan-Kadle AA, Osman AM, Ibrahim AM, Mohamed AA, de Oliveira CJB, Vieira RFC. One Health in Somalia: Present status, opportunities, and challenges. One Health. 2023 Dec 21;18:100666. doi:10.1016/j.onehlt.2023.100666 PubMed PMID: 38226139; PubMed Central PMCID: PMC10788489.

85. Hegewisch-Taylor J, Dreser A, Aragón-Gama AC, Moreno-Reynosa MA, Ramos Garcia C, Ruckert A, et al. Analyzing One Health governance and implementation challenges in Mexico. Glob Public Health. 2024 Dec 31;19(1):2377259. doi:10.1080/17441692.2024.2377259

86. Hein W, Aglanu LM, Mensah-Sekyere M, Harant A, Brinkel J, Lamshöft M, et al. Fighting Antimicrobial Resistance: Development and Implementation of the Ghanaian National Action Plan (2017–2021). Antibiotics. 2022 May;11(5):5. doi:10.3390/antibiotics11050613

87. Hoque R, Ahmed SM, Naher N, Islam MA, Rousham EK, Islam BZ, et al. Tackling antimicrobial resistance in Bangladesh: A scoping review of policy and practice in human, animal and environment sectors. PLOS ONE. 2020 Jan 27;15(1):e0227947. doi:10.1371/journal.pone.0227947

88. Humboldt-Dachroeden S. Translating One Health knowledge across different institutional and political contexts in Europe. One Health Outlook. 2023;5(1):1. doi:10.1186/s42522-022-00074-x

89. Igihozo G, Henley P, Ruckert A, Karangwa C, Habimana R, Manishimwe R, et al. An environmental scan of one health preparedness and response: the case of the Covid-19 pandemic in Rwanda. One Health Outlook. 2022 Dec;4(1):2. doi:10.1186/s42522-021-00059-2

90. Innes GK, Lambrou AS, Thumrin P, Thukngamdee Y, Tangwangvivat R, Doungngern P, et al. Enhancing global health security in Thailand: Strengths and challenges of initiating a One Health approach to avian influenza surveillance. One Health. 2022;14:100397. doi:10.1016/j.onehlt.2022.100397

91. IACG. No time to Wait: Securing the future from drug-resistant infections Summary of Recommendations and Key Messages [Internet]. 2019 [cited 2024 May 17]. Available from: https://cdn.who.int/media/docs/default-source/antimicrobial-resistance/amr-gcp-tjs/iacg/summaries/iacg_final_summary_en.pdf?sfvrsn=f346e650_5

92. Iwu CD, Patrick SM. An insight into the implementation of the global action plan on antimicrobial resistance in the WHO African region: A roadmap for action. Int J Antimicrob Agents. 2021 Oct;58(4):106411. doi:10.1016/j.ijantimicag.2021.106411

93. Johnson I, Hansen A, Bi P. The challenges of implementing an integrated One Health surveillance system in Australia. Zoonoses Public Health. 2018;65(1):e229–36. doi:10.1111/zph.12433

94. Joshi MP, Hafner T, Twesigye G, Ndiaye A, Kiggundu R, Mekonnen N, et al. Strengthening multisectoral coordination on antimicrobial resistance: a landscape analysis of efforts in 11 countries. J Pharm Policy Pract. 2021 Feb 28;14(1):27. doi:10.1186/s40545-021-00309-8

95. Khan MS, Durrance-Bagale A, Mateus A, Sultana Z, Hasan R, Hanefeld J. What are the barriers to implementing national antimicrobial resistance action plans? A novel mixed-methods policy analysis in Pakistan. Health Policy Plan. 2020;35(8):973–82. doi:10.1093/heapol/czaa065

96. Kimani T, Kiambi S, Eckford S, Njuguna J, Makonnen Y, Rugalema G, et al. Expanding beyond zoonoses: the benefits of a national One Health coordination mechanism to address antimicrobial resistance and other shared health threats at the human-animal-environment interface in Kenya. Rev Sci Tech Int Off Epizoot. 2019;38(1):155–71. doi:10.20506/rst.38.1.2950

97. Koduah A, Gyansa-Lutterodt M, Hedidor G, Sekyi-Brown R, Asiedu-Danso M, Asare B, et al. Antimicrobial resistance national level dialogue and action in Ghana: setting and sustaining the agenda and outcomes. ONE Health OUTLOOK. 2021;3(1). Located at: WOS:000708452600001. doi:10.1186/s42522-021-00051-w

98. Kolla RA, Lokugalappatti LGS, Clark DA, Brook RK. Enhancing inter-organizational collaboration for wildlife disease surveillance in Sri Lanka. Zoonoses Public Health. 2022;69(7):792–805. doi:10.1111/zph.12969

99. Lâm S, Dang-Xuan S, Bekele M, Amenu K, Alonso S, Unger F, et al. How do food safety technical working groups within a One Health framework work? Experiences from Vietnam and Ethiopia. One Health Outlook. 2024;6(1):16. doi:10.1186/s42522-024-00110-y

100. Li H, Chen Y, Machalaba CC, Tang H, Chmura AA, Fielder MD, et al. Wild animal and zoonotic disease risk management and regulation in China: Examining gaps and One Health opportunities in scope, mandates, and monitoring systems. One Health. 2021;13. doi:10.1016/j.onehlt.2021.100301

101. Government of South Australia, World Health Organization, Kickbusch I. Progressing the Sustainable Development Goals through Health in All Policies: Case studies from around the world. Adel Gov S Aust. 2017.

102. Lioupi O, Kostoulas P, Monti G, Griva K, Billinis C, Tsiamis C. Harnessing Medical Bioethics Mediation to Advance One Health Governance. Vet Sci. 2025 Dec 20;13(1):8. doi:10.3390/vetsci13010008

103. Lokossou VK, Atama NC, Nzietchueng S, Koffi BY, Iwar V, Oussayef N, et al. Operationalizing the ECOWAS regional one health coordination mechanism (2016-2019): Scoping review on progress, challenges and way forward. One Health. 2021;13:100291. doi:10.1016/j.onehlt.2021.100291

104. Lota MMM, Chua AQ, Azupardo K, Lumangaya C, Reyes KAV, Villanueva SYAM, et al. A Qualitative Study on the Design and Implementation of the National Action Plan on Antimicrobial Resistance in the Philippines. Antibiot Basel Switz. 2022 Jun 17;11(6):820. doi:10.3390/antibiotics11060820 PubMed PMID: 35740226; PubMed Central PMCID: PMC9220349.

105. Lowe R, Ryan SJ, Mahon R, Van Meerbeeck CJ, Trotman AR, Boodram LG, et al. Building resilience to mosquito-borne diseases in the Caribbean. PLoS Biol. 2020;18(11):e3000791. doi:10.1371/journal.pbio.3000791

106. McKenzie JS, Dahal R, Kakkar M, Debnath N, Rahman M, Dorjee S, et al. One Health research and training and government support for One Health in South Asia. Infect Ecol Epidemiol. 2016 Nov 29;6:10.3402/iee.v6.33842. doi:10.3402/iee.v6.33842 PubMed PMID: 27906123; PubMed Central PMCID: PMC5131453.

107. McPake B, Gilbert K, Vong S, Ros B, Has P, Khuong AT, et al. Role of regulatory capacity in the animal and human health systems in driving response to zoonotic disease outbreaks in the the Mekong region. One Health. 2022;14. doi:10.1016/j.onehlt.2022.100369

108. McPherson A, Hill PS, Kama M, Reid S. Exploring governance for a One Health collaboration for leptospirosis prevention and control in Fiji: Stakeholder perceptions, evidence, and processes. Int J Health Plann Manage. 2018;33(3):677–89. doi:10.1002/hpm.2521

109. Mhone AL, Muloi DM, Moodley A. One Health: governance and regulatory framework for antimicrobial use in Malawi. Sci One Health. 2025;4:100119. doi:10.1016/j.soh.2025.100119

110. Mickelsson M, Oljans E. Agile policies for antimicrobial resistance: A contextual approach to sustainable health challenges. Glob Public Health. 2025 Dec 31;20(1):2522913. doi:10.1080/17441692.2025.2522913

111. Mitchell MEV, Alders R, Unger F, Nguyen-Viet H, Le TTH, Toribio JA. The challenges of investigating antimicrobial resistance in Vietnam - what benefits does a One Health approach offer the animal and human health sectors? BMC Public Health. 2020 Feb 11;20(1):213. doi:10.1186/s12889-020-8319-3

112. Molina-Flores B, Vigilato MAN, Rocha F, Cossivi O, Corrales M, Vásquez Niño GA, et al. Assessment of One Health Initiatives from a Veterinary Public Health Approach in Latin America and the Caribbean. Trop Med Infect Dis. 2025 Nov 6;10(11):315. doi:10.3390/tropicalmed10110315

113. Mor N. Organising for One Health in a developing country. One Health. 2023;17. doi:10.1016/j.onehlt.2023.100611

114. Moura P, Borck Høg B, Alban L, Sönksen UW, Ribeiro Duarte AS, Sandberg M. Evaluating the OH-EpiCap tool using the Danish integrated surveillance program for AMU and AMR as a case study. Front Public Health. 2023;11. doi:10.3389/fpubh.2023.1127701

115. Mphande-Nyasulu FA, Yap NJ, Teo CH, Chang LY, Tay ST. Outbreak preparedness and response strategies in ASEAN member states: a scoping review. IJID Reg. 2024;12. doi:10.1016/j.ijregi.2024.100430

116. Mulenga GM, Namangala B, Chilongo K, Henning L, Gummow B. Policy and Linkages in the Application of a One Health System for Reporting and Controlling African Trypanosomiasis and Other Zoonotic Diseases in Zambia. Pathogens. 2021;11(1). doi:10.3390/pathogens11010030

117. Munyua PM, Njenga MK, Osoro EM, Onyango CO, Bitek AO, Mwatondo A, et al. Successes and challenges of the One Health approach in Kenya over the last decade. BMC Public Health. 2019;19(Suppl 3):465. doi:10.1186/s12889-019-6772-7

118. Mwacalimba KK, Green J. One health and development priorities in resource-constrained countries: Policy lessons from avian and pandemic influenza preparedness in Zambia. Health Policy Plan. 2015;30(2):215–22. doi:10.1093/heapol/czu001

119. Nair M, Zeegers MP, Varghese GM, Burza S. India’s National Action Plan on Antimicrobial Resistance: a critical perspective. J Glob Antimicrob Resist. 2021;27:236–8. doi:10.1016/j.jgar.2021.10.007

120. Nana SD, Caffin JH, Duboz R, Antoine-Moussiaux N, Binot A, Diagbouga PS, et al. Towards an integrated surveillance of zoonotic diseases in Burkina Faso: the case of anthrax. BMC Public Health. 2022;22(1). doi:10.1186/s12889-022-13878-3

121. Nana SD, Duboz R, Diagbouga PS, Hendrikx P, Bordier M. A participatory approach to move towards a One Health surveillance system for anthrax in Burkina Faso. PLoS ONE. 2024;19(6 June). doi:10.1371/journal.pone.0304872

122. Nantima N, Ilukor J, Kaboyo W, Ademun ARO, Muwanguzi D, Sekamatte M, et al. The importance of a One Health approach for prioritising zoonotic diseases to focus on capacity-building efforts in Uganda. Rev Sci Tech. 2019;38(1):315–25. doi:10.20506/rst.38.1.2963

123. Nguendo-Yongsi B, Muzenda T, Feudjio YBD, Momo DNK, Oni T. Intersectoral collaboration for healthier human settlements: perceptions and experiences from stakeholders in Douala, Cameroon. Cities Health. 2022;6(3):602–15. doi:10.1080/23748834.2022.2078071

124. Nguyen HTT, Afriyie DO, Tran CH, Dang AD, Tran DN, Dang TQ, et al. Progress towards rabies control and elimination in Vietnam. Rev Sci Tech. 2019;38(1):199–212. doi:10.20506/rst.38.1.2953

125. Nguyen-Viet H, Lam S, Nguyen-Mai H, Trang DT, Phuong VT, Tuan NDA, et al. Decades of emerging infectious disease, food safety, and antimicrobial resistance response in Vietnam: The role of One Health. One Health. 2022;14. doi:10.1016/j.onehlt.2021.100361

126. Nyokabi NS, Moore H, Berg S, Lindahl J, Phelan L, Gimechu G, et al. Implementing a one health approach to strengthen the management of zoonoses in Ethiopia. One Health. 2023 Jun;16:100521. doi:10.1016/j.onehlt.2023.100521

127. Letelier JJT, OIE. Strengthening Veterinary Services through the OIE PVS Pathway. 2019.

128. Okello AL, Bardosh K, Smith J, Welburn SC. One Health: past successes and future challenges in three African contexts. PLoS Negl Trop Dis. 2014;8(5):e2884. doi:10.1371/journal.pntd.0002884

129. Okello A, Welburn S, Smith J. Crossing institutional boundaries: mapping the policy process for improved control of endemic and neglected zoonoses in sub-Saharan Africa. Health Policy Plan. 2015 Jul 1;30(6):804–12. doi:10.1093/heapol/czu059

130. Okia M, Okui P, Lugemwa M, Govere JM, Katamba V, Rwakimari JB, et al. Consolidating tactical planning and implementation frameworks for integrated vector management in Uganda. Malar J. 2016;15(1). doi:10.1186/s12936-016-1269-7

131. Osman AY, Saidouni A, Wambua LW, Mahrous H, Malik SMMR, Lubogo M, et al. IHR-PVS National Bridging Workshop for Somalia: An interactive and participatory approach for operationalizing the One Health roadmap. One Health. 2024;19:100858. doi:10.1016/j.onehlt.2024.100858

132. Parodi P, Schmid G, Ward D. One health in central Asia. A situational analysis informing the future. G Ital Med Trop. 2011;16(3–4):23–37.

133. Qiu Y, Ferreira JP, Ullah RW, Flanagan P, Zaheer MU, Tahir MF, et al. Assessment of the Implementation of Pakistan’s National Action Plan on Antimicrobial Resistance in the Agriculture and Food Sectors. Antibiot Basel Switz. 2024 Feb 22;13(3):206. doi:10.3390/antibiotics13030206 PubMed PMID: 38534641; PubMed Central PMCID: PMC10967498.

134. Rubin O, Nyanda SS, Norström M. One health rapid qualitative assessment: Exploring local governance gaps in Tanzania. One Health. 2026 Jun;22:101353. doi:10.1016/j.onehlt.2026.101353

135. Rubin C, Dunham B, Sleeman J. Making One Health a Reality--Crossing Bureaucratic Boundaries. Microbiol Spectr. 2014;2(1):OH-0016. doi:10.1128/microbiolspec.OH-0016-2012

136. Sande S, Zimba M, Nyasvisvo D, Mukuzunga M, Kooma EH, Mberikunashe J, et al. Getting ready for integrated vector management for improved disease prevention in Zimbabwe: a focus on key policy issues to consider. Malar J. 2019;18(1):322. doi:10.1186/s12936-019-2965-x

137. Schneider H, Barisic N, Batalha AI de V, Quinet EF. OIE PVS Evaluation Report of the Veterinary Services of Brazil: 10 - 28 February 2014. World Organisation for Animal Health (OIE); 2014.

138. Shabangu K, Essack SY, Duma SE. Policy Makers’ Perceptions on Implementation of National Action Plans on Antimicrobial Resistance in South Africa and Eswatini Using Coordination, Accountability, Resourcing, Regulation and Ownership Framework (2018–2019). Antibiotics. 2025 Jul 11;14(7):696. doi:10.3390/antibiotics14070696

139. Shabangu K, Essack SY, Duma SE. Barriers to Implementing National Action Plans on Antimicrobial Resistance using a One Health Approach: Policy-Makers’ Perspectives from South Africa and Eswatini. J Glob Antimicrob Resist. 2023 Feb 28. doi:10.1016/j.jgar.2023.02.007

140. Shiferaw ML, Doty JB, Maghlakelidze G, Morgan J, Khmaladze E, Parkadze O, et al. Frameworks for Preventing, Detecting, and Controlling Zoonotic Diseases. Emerg Infect Dis. 2017;23(13):S71-6. doi:10.3201/eid2313.170601

141. Smith J, Taylor EM, Kingsley P. One World-One Health and neglected zoonotic disease: Elimination, emergence and emergency in Uganda. Soc Sci Med. 2015;129:12–9. doi:10.1016/j.socscimed.2014.06.044

142. Sow MM, Delpy L, Ciss M, Fall AG, Diouf ND, Caffin JH, et al. Governing antibiotic resistance through One Health: Insights from the political and legal landscape in Senegal. Sulis G, editor. PLOS Glob Public Health. 2026 Mar 12;6(3):e0005889. doi:10.1371/journal.pgph.0005889

143. Sow MM, Ciss M, Diouf ND, Fall AG, Dia NM, Penney T, et al. Supporting One Health policies to manage antibiotic resistance in Senegal: a systems analysis using group model building. Front Public Health. 2025 Nov 25;13:1689609. doi:10.3389/fpubh.2025.1689609

144. Standley CJ, Carlin EP, Sorrell EM, Barry AM, Bile E, Diakite AS, et al. Assessing health systems in Guinea for prevention and control of priority zoonotic diseases: A One Health approach. One Health. 2019;7. doi:10.1016/j.onehlt.2019.100093

145. Stewart-Ibarra AM, Romero M, Hinds AQJ, Lowe R, Mahon R, Van Meerbeeck CJ, et al. Co-developing climate services for public health: Stakeholder needs and perceptions for the prevention and control of Aedes-transmitted diseases in the Caribbean. PLoS Negl Trop Dis. 2019;13(10):e0007772. doi:10.1371/journal.pntd.0007772

146. Sumpradit N, Wongkongkathep S, Malathum K, Janejai N, Paveenkittiporn W, Yingyong T, et al. Thailand’s national strategic plan on antimicrobial resistance: progress and challenges. Bull World Health Organ. 2021 Sep 1;99(9):661–73. doi:10.2471/BLT.20.280644

147. Tangcharoensathien V, Sattayawutthipong W, Kanjanapimai S, Kanpravidth W, Brown R, Sommanustweechai A. Antimicrobial resistance: From global agenda to national strategic plan, Thailand. Bull World Health Organ. 2017;95(8):599–603. doi:10.2471/BLT.16.179648

148. Tegegne HA, Freeth FTA, Bogaardt C, Taylor E, Reinhardt J, Collineau L, et al. Implementation of One Health surveillance systems: Opportunities and challenges - lessons learned from the OH-EpiCap application. One Health Amst Neth. 2024 Jun;18:100704. doi:10.1016/j.onehlt.2024.100704 PubMed PMID: 38496337; PubMed Central PMCID: PMC10940803.

149. Tiensin T, Chuxnum T. How can we progress the cooperation between animal health sector and public health sector?

150. Time MS, Veggeland F. From ideal to reality: governance of AMR in a multi-level setting. J Eur Public Policy. 2024;31(12):4063–87. doi:10.1080/13501763.2024.2400271

151. Travis DA, Chapman DW, Craft ME, Deen J, Farnham MW, Garcia C, et al. One Health: Lessons Learned from East Africa. Microbiol Spectr. 2014;2(1):OH-0017. doi:10.1128/microbiolspec.OH-0017-2012

152. Valenzuela S, Lao PE, Apostol GLC, Conda LEA, Dayapera LZA, Enriquez AB, et al. Situational analysis of antimicrobial resistance policies and program implementation in the Philippines, 2019–2023. One Health. 2025 Dec;21:101255. doi:10.1016/j.onehlt.2025.101255

153. Wakimoto MD, Menezes RC, Pereira SA, Nery T, Castro-Alves J, Penetra SLS, et al. COVID-19 and zoonoses in Brazil: Environmental scan of one health preparedness and response. One Health. 2022;14. doi:10.1016/j.onehlt.2022.100400

154. Wallinga D, Smit LAM, Davis MF, Casey JA, Nachman KE. A Review of the Effectiveness of Current US Policies on Antimicrobial Use in Meat and Poultry Production. Curr Environ Health Rep. 2022;9(2):339–54. doi:10.1007/s40572-022-00351-x

155. Weaver J, Facelli P. OIE PVS Evaluation Follow-Up Mission Report: Rwanda. World Organisation for Animal Health (OIE); 2019.

156. Wignjadiputro I, Widaningrum C, Setiawaty V, Widuri Wulandari E, Sihombing S, Prasetyo WA, et al. Whole-of-society approach for influenza pandemic epicenter Containment exercise in Indonesia. J Infect Public Health. 2020;13(7):994–7. doi:10.1016/j.jiph.2019.12.009

157. Woolaston K, Nay Z, Baker ML, Brockett C, Bruce M, Degeling C, et al. An argument for pandemic risk management using a multidisciplinary One Health approach to governance: an Australian case study. Glob Health. 2022;18(1). doi:10.1186/s12992-022-00850-4

158. World Bank, FAO. Reducing Pandemic Risks at Source: Wildlife, Environment and One Health Foundations in East and South Asia [Internet]. Washington, DC; 2022 [cited 2025 Jul 9]. Available from: https://doi.org/10.1596/37327

159. World Bank and the World Health Organization. Sustaining Action Against Antimicrobial Resistance: A Case Series of Country Experiences. Washington, DC; 2022.

160. Organization WH. Tackling antimicrobial resistance (AMR) together: working paper 1.0: multisectoral coordination. World Health Organization; 2018.

161. World Health Organization. WHO implementation handbook for national action plans on antimicrobial resistance [Internet]. World Health Organization; 2022. Available from: https://iris.who.int/bitstream/handle/10665/352204/9789240041981-eng.pdf?sequence=1

162. Resource mobilisation for AMR: Getting AMR into plans and budgets of government and development partners: Nepal country report.

163. WHO, OIE. WHO-OIE operational framework for Good governance at the human-animal interface: Bridging WHO and OIE tools for the assessment of national capacities [Internet]. 2014 [cited 2022 Jun 3]. Available from: https://www.woah.org/app/uploads/2021/03/who-oie-operational-framework-final2.pdf

164. The Independent Oversight and Advisory Committee (IOAC) for the WHO Health Emergencies Programme, Harvey F, Konyndyk. Viet Nam Mission Report: 2 May 2018 - 4 May 2018. 2018.

165. Yambayamba MK, Kazadi EK, Ayumuna BM, Kapepula PM, Kalemayi MN, Kangudie DM, et al. Learning from over ten years of implementing the One Health approach in the Democratic Republic of Congo: A qualitative study. One Health. 2024 Dec 1;19:100934. doi:10.1016/j.onehlt.2024.100934

166. Yang D, Dyar OJ, Yin J, Ma W, Sun Q, Lundborg CS. Antimicrobial resistance in China across human, animal, and environment sectors - a review of policy documents using a governance framework. Lancet Reg Health West Pac. 2024;48(101774968):101111. Located at: Ovid MEDLINE(R) PubMed-not-MEDLINE. doi:10.1016/j.lanwpc.2024.101111

167. Yasobant S, Bruchhausen W, Saxena D, Falkenberg T. ‘One Health’ Actors in Multifaceted Health Systems: An Operational Case for India. Healthc Switz. 2020;8(4). doi:10.3390/healthcare8040387

168. Yasobant S, Bruchhausen W, Saxena D, Falkenberg T. Systemic factors for enhancing intersectoral collaboration for the operationalization of One Health: a case study in India. Health Res Policy Syst. 2021;19(1):75. doi:10.1186/s12961-021-00727-9

169. Yopa DS, Massom DM, Kiki GM, Sophie RW, Fasine S, Thiam O, et al. Barriers and enablers to the implementation of one health strategies in developing countries: a systematic review. Front Public Health. 2023 Nov 23;11:1252428. doi:10.3389/fpubh.2023.1252428 PubMed PMID: 38074697; PubMed Central PMCID: PMC10701386.

170. Zaidi MB, Dreser A, Figueroa IM. A collaborative initiative for the containment of antimicrobial resistance in Mexico. Zoonoses Public Health. 2015;62 Suppl 1:52–7. doi:10.1111/zph.12166

171. Zhang Q yu, Zhang YY, Liu JS, Li XC, Zhu ZL, Feng XY, et al. Integrating One Health governance in China: Assessing structural implementation and operational entry points. One Health. 2025 Dec;21:101209. doi:10.1016/j.onehlt.2025.101209
