# Supplementary material for: Implementing One Health governance approaches to mitigate antimicrobial resistance across institutional, social, economic and political contexts: a scoping review
Source: BMJ Open. 2026 Jul 8;16(7):e115471. doi: 10.1136/bmjopen-2025-115471 (PMC13347904; doi:10.1136/bmjopen-2025-115471)
Supplement: Supplementary file 6 [file bmjopen-2025-115471.reviewer_comments.pdf]

PEER REVIEW HISTORY

BMJ Open publishes all reviews undertaken for accepted manuscripts. Reviewers are asked to complete a checklist review form and are provided with free text boxes to elaborate on their assessment. These free text comments are reproduced below.

ARTICLE DETAILS

Title (Provisional)

Implementing One Health governance approaches to mitigate antimicrobial resistance across institutional, social, economic, and political contexts: A scoping review

Authors

Clifford Astbury, Chloe; Hecker, Taylor; Singh, Jaskeerat; Naro, Suzanne Garkay; Wallace, Lauren; Boily-Larouche, Geneviève; Poirier, Mathieu J; Ahmed, Yahaya Ali; Balachandran, Anand; Dobreva, Zlatina; Fuller, Walter; Gahembare, Laetitia; Mazengiya, Yidnekachew Degefaw; Zayed, Bassim; Ruckert, Arne

---

VERSION 1 - REVIEW

**Reviewer**	**1**
**Name**	**Markwart, Robby**
**Affiliation**	**Jena University Hospital, Institute of General Practice and Family Medicine**
**Date**	**11-Feb-2026**
**COI**	**None**

The study titled 'Implementing One Health governance approaches to mitigate antimicrobial resistance across institutional, social, economic, and political contexts: A systematic scoping review Authors' investigated the design and implementation of one health governance in different settings by the means of a systematic scoping review. It aimed to map and synthesise evidence on context-specific approaches to one health governance and their performance, to inform effective governance design across settings. Overall, the articles concludes that there is a wide spectrum of one health governance approaches and different country-aspects shapes their implementation. However, evidence on the effectiveness of different approaches is limited.

Comments:

• Overall, the systematic scoping review is well conducted, and the results are clearly presented.

• A major limitation of the article is the restriction to publications in English and French. This is surprising given the participation of WHO representatives from multiple world regions among the authors. Many governance-related documents are published only in national or official languages, making it likely that relevant evidence on one health governance approaches was not captured. For example, in Germany, the national one health governance approach, the "DART 2030 Deutsche Antibiotika-Resistenzstrategie" is published only in German. Although the authors briefly acknowledge this limitation in the Discussion, its potential implications for the completeness of the evidence base may warrant further consideration.

• The article focuses on institutional, social, economic, and political contexts; however, for clarity, the authors could more explicitly define what is meant by "context" and provide examples of these different contextual dimensions.

• "In this review, we identified a range of contextual factors that shape One Health governance, One Health governance mechanisms, and conceptions of effective One Health governance (Figure 2)." This statement should not be part of the result and should be moved to the introduction.

• Table 2 includes several abbreviations that are not defined (e.g. SIDA, NORAD, IDRC). Abbreviations should be explained at first mention, either in the table or in a corresponding footnote.

• The statement "Additional causal, mechanistic evidence based on comparative analysis and in-depth case studies would support evidence-informed decision-making" is quite general. The paragraph would be strengthened if the authors more clearly specified which types of evidence are currently lacking and outlined how future studies could be concretely designed to address these gaps (e.g., governance interventions or models, comparators, and relevant outcomes).

• While the discussion effectively maps contextual variation, parts read as extended summaries of findings rather than higher-level synthesis. Consider more explicitly describing why certain governance mechanisms work better in specific contexts (e.g. political economy, institutional path dependence, decentralisation theory), rather than primarily what was observed.

• The discussion of low- and lower middle-income countries is more detailed than that of upper middle- and high-income countries. This reflects the evidence base, it would be helpful to explicitly acknowledge this imbalance as a feature of the literature, and/or slightly expand the discussion to include more concrete examples or governance mechanisms beyond resourcing and private-sector influence.

• Some themes are mentioned multiple times (e.g. sustainability of funding, donor influence, political will). These could be consolidated to improve flow and reduce length.

**Reviewer**	**2**
**Name**	**Depuydt , Pieter**
**Affiliation**	**Ghent University Hospital, Department of Intensive Care Medicine**
**Date**	**17-Mar-2026**
**COI**	**None**

Dr. Clifford Astbury and colleagues present a scoping review examining the design and implementation of "One Health" governance across national, regional, and international contexts. The manuscript analyzes governance design features, implementation strategies, evidence of effectiveness, and perceived facilitators and barriers. To structure a heterogeneous body of literature, the authors apply an a priori framework comprising six analytical dimensions. The review incorporates systematic searches of PubMed, Scopus, and Web of Science in accordance with PRISMA-ScR guidance, complemented by a grey literature search across ten organizational websites.

The research questions are clearly formulated, and the search strategy is transparent and methodologically sound. The authors should be commended for their effort to impose analytical structure on a complex and diverse evidence base. This approach enables the identification of cross-cutting patterns and generalizable insights. The manuscript is well organized and clearly written. A key strength lies in its provision of a contemporary, state-of-the-art overview of an expansive literature, effectively serving as an accessible entry point for researchers and policymakers engaging with One Health governance.

A notable limitation, however, is the likely omission of relevant documents despite the breadth of the search strategy. For example, the report "Proposals for a More Effective Antibiotic Policy in Belgium" by the Belgian Health Care Knowledge Centre—a federal scientific institute—addresses all six analytical dimensions of a national 'One Health' concept outlined in the review but does not appear to have been captured (see attached file). Based on the reported search strategy, this omission is plausible and raises concerns about the potential exclusion of other pertinent national-level governance reports. Greater reflection on the completeness of grey literature retrieval, and potential strategies to mitigate such gaps, would still strengthen the manuscript.

Minor comment: Page 2: We conducted a systematic scoping review, searching PubMed, Scopus, Web of Science, and grey literature sources in December 2024: To avoid eventual confusion between the terms 'systematic review' and 'scoping review', I would suggest the phrasing: 'systematically conducted scoping review'

VERSION 1 - AUTHOR RESPONSE

Reviewer: 1
Dr. Robby Markwart, Jena University Hospital
Comments to the Author:
The study titled 'Implementing One Health governance approaches to mitigate antimicrobial resistance across institutional, social, economic, and political contexts: A systematic scoping review Authors' investigated the design and implementation of one health governance in different settings by the means of a systematic scoping review. It aimed to map and synthesise evidence on context-specific approaches to one health governance and their performance, to inform effective governance design across settings. Overall, the articles concludes that there is a wide spectrum of one health governance approaches and different country-aspects shapes their implementation. However, evidence on the effectiveness of different approaches is limited.

Comments:

• Overall, the systematic scoping review is well conducted, and the results are clearly presented.

Thank you very much!

• A major limitation of the article is the restriction to publications in English and French. This is surprising given the participation of WHO representatives from multiple world regions among the authors. Many governance-related documents are published only in national or official languages, making it likely that relevant evidence on one health governance approaches was not captured. For example, in Germany, the national one health governance approach, the "DART 2030 Deutsche Antibiotika-Resistenzstrategie" is published only in German. Although the authors briefly acknowledge this limitation in the Discussion, its potential implications for the completeness of the evidence base may warrant further consideration.

We thank the reviewer for raising this important point as we agree that limiting the search to English and French may have led to the omission of relevant governance documents published exclusively in other languages. We have added additional reflections in the limitations section on the implications of this. We now note that this approach may have introduced a degree of selection bias since in the domain of One Health governance policy documents and implementation frameworks are often produced in national or official languages and not always translated into English or French. This may

have led to the underrepresentation of institutional mechanisms or governance innovations that are not captured in the Anglo- or Francophone peer-reviewed and grey literature (page 26-27, line 634-6450 (marked copy) / page 26-27, line 609-622 (clean copy)).

• The article focuses on institutional, social, economic, and political contexts; however, for clarity, the authors could more explicitly define what is meant by "context" and provide examples of these different contextual dimensions.

Thank you for this recommendation, we have added a sentence in the methods section where we now more clearly define context as the variation in institutional arrangements, actor configurations, power relations, and socio-economic and political conditions within which governance processes are embedded and enacted (Page 8, line 208-211 (marked copy) / page 8, line 186-189 (clean copy))

• "In this review, we identified a range of contextual factors that shape One Health governance, One Health governance mechanisms, and conceptions of effective One Health governance (Figure 2)." This statement should not be part of the result and should be moved to the introduction.

This statement introduces the framework that we created based on the results of our study analysis. We changed the wording to "Through our analysis, we identified..." to increase clarity. (Page 9, line 248 (marked copy) / Page 9, line 226 (clean copy)

• Table 2 includes several abbreviations that are not defined (e.g. SIDA, NORAD, IDRC). Abbreviations should be explained at first mention, either in the table or in a corresponding footnote.

Thank you for noting this oversight. We have reviewed the table and spelled out abbreviations at first use if outside of Table 2 and expanded on abbreviations under Table 2 if not mentioned elsewhere.

• The statement "Additional causal, mechanistic evidence based on comparative analysis and in-depth case studies would support evidence-informed decision-making" is quite general. The paragraph would be strengthened if the authors more clearly specified which types of evidence are currently lacking and outlined how future studies could be concretely designed to address these gaps (e.g., governance interventions or models, comparators, and relevant outcomes).

Thank you for this recommendation, we agree, and added a new sentence that highlights how qualitative comparative analysis and case study research based on key informant interviews and document analysis can be used to advance further knowledge by filling existing knowledge gaps, such as lack of understanding of the configurational conditions of successful One Health governance (page 26, line 620-625 (marked copy) / page 26, line 595-600 (clean copy)) and policymaker knowledge of context-specific governance strategies.

• While the discussion effectively maps contextual variation, parts read as extended summaries of findings rather than higher-level synthesis. Consider more explicitly describing why certain governance mechanisms work better in specific contexts (e.g. political economy, institutional path dependence, decentralisation theory), rather than primarily what was observed.

Given that much of the reviewed literature does not causally connect specific governance conditions to specific outcomes, we are not able to systematically assess and theoretically reflect on what specific mechanisms lead to what specific outcomes. However, we have expanded the discussion section to provide additional reflections and connect better with the existing theoretical literature in this area (page 24, line 539-544 (marked copy) / page 24, line 515-520 (clean copy)). We also want to note that this scoping review was used to design and run a qualitative comparative analysis (QCA) in the software package R to link specific governance dimensions and mechanisms to specific AMR One Health governance outcomes. The findings from this analysis will be submitted for publication soon and will throw additional light on the question of the reviewer.

• The discussion of low- and lower middle-income countries is more detailed than that of upper middle- and high-income countries. This reflects the evidence base, it would be helpful to explicitly acknowledge this imbalance as a feature of the literature, and/or slightly expand the discussion to include more concrete examples or governance mechanisms beyond resourcing and private-sector influence.

We strongly agree with the reviewer's point about the need to acknowledge the unbalanced nature of the literature and have integrated this into our reworded limitations section where we now note that our evidence is largely based on findings from LMICs (page 27, line 648-650 (marked copy) / page 27, line 622-625).

• Some themes are mentioned multiple times (e.g. sustainability of funding, donor influence, political will). These could be consolidated to improve flow and reduce length.

We have reviewed the manuscript for flow and aimed to reduce redundancies where possible, as suggested. However, since various aspects (e.g. financial sustainability) are discussed in different contexts, repetition cannot always be avoided.

Reviewer: 2
Prof. Pieter Depuydt , Ghent University Hospital
Comments to the Author:
Dr. Clifford Astbury and colleagues present a scoping review examining the design and implementation of "One Health" governance across national, regional, and international contexts. The manuscript analyzes governance design features, implementation strategies, evidence of effectiveness, and perceived facilitators and barriers. To structure a heterogeneous body of literature, the authors apply an a priori framework comprising six analytical dimensions. The review incorporates systematic searches of PubMed, Scopus, and Web of Science in accordance with PRISMA-ScR guidance, complemented by a grey literature search across ten organizational websites.

The research questions are clearly formulated, and the search strategy is transparent and methodologically sound. The authors should be commended for their effort to impose analytical structure on a complex and diverse evidence base. This approach enables the identification of cross-cutting patterns and generalizable insights. The manuscript is well organized and clearly written. A key strength lies in its provision of a contemporary, state-of-the-art overview of an expansive literature, effectively serving as an accessible entry point for researchers and policymakers engaging with One Health governance.

Thank you very much for this very positive assessment.

A notable limitation, however, is the likely omission of relevant documents despite the breadth of the search strategy. For example, the report "Proposals for a More Effective Antibiotic Policy in Belgium" by the Belgian Health Care Knowledge Centre—a federal scientific institute—addresses all six analytical dimensions of a national 'One Health' concept outlined in the review but does not appear to have been captured (see attached file). Based on the reported search strategy, this omission is plausible and raises concerns about the potential exclusion of other pertinent national-level governance reports. Greater reflection on the completeness

of grey literature retrieval, and potential strategies to mitigate such gaps, would still strengthen the manuscript.

We appreciate the reviewer's feedback and agree that focusing predominantly on global and regional level grey literature, published in English and French, has introduced important limitations to our analysis. During the revision process, we prioritized rescreening of the academic article base to ensure that the academic literature is fully up to date. However, we have expanded our limitation section to provide greater reflection on what this limited linguistic scope of the grey literature inclusion implies. We now notice that language and scope limitations of the grey literature screening may have limited insights into national level idiosyncrasies that would have been captured with a broader search strategy, and recommend for future research in this area to use AI-based translation tools that are now available which were not as reliable when we started this research project.

Minor comment: Page 2: We conducted a systematic scoping review, searching PubMed, Scopus, Web of Science, and grey literature sources in December 2024: To avoid eventual confusion between the terms 'systematic review' and 'scoping review', I would suggest the phrasing: 'systematically conducted scoping review'

We agree and have reworded to ' systematically conducted scoping review" throughout the manuscript and changed the title.

Reviewer: 1
If you have selected 'Yes' above, please provide details of any competing interests.: Not applicable

Reviewer: 2
If you have selected 'Yes' above, please provide details of any competing interests.: Not applicable

---

VERSION 2 - REVIEW

**Reviewer**	**1**
**Name**	**Markwart, Robby**
**Affiliation**	**Jena University Hospital, Institute of General Practice and Family Medicine**
**Date**	**07-May-2026**

COI

I am satisfied with the revision.

**Reviewer**	**2**
**Name**	**Depuydt , Pieter**
**Affiliation**	**Ghent University Hospital, Department of Intensive Care Medicine**
**Date**	**19-May-2026**
**COI**	

The authors have clearly addressed the limitations of the study. I understand that expanding the literature review to documents in official national languages other than English and French should be done without bias, thus introducing the requirement to translate from a broad, even, global range of languages, which is currently beyond the cope and practical feasibility.
